# Representing number in the real-time processing of agreement: self-paced reading evidence from Arabic

**DOI:** 10.3389/fpsyg.2015.00347

**Published:** 2015-04-09

**Authors:** Matthew A. Tucker, Ali Idrissi, Diogo Almeida

**Affiliations:** ^1^Language, Mind, and Brain Laboratory, Science Division, Department of Psychology, New York University Abu DhabiAbu Dhabi, UAE; ^2^Department of English Literature and Linguistics, Qatar UniversityDoha, Qatar

**Keywords:** working memory, agreement, plurals, abstract morphology, self-paced reading, Arabic, sentence processing

## Abstract

In the processing of subject-verb agreement, non-subject plural nouns following a singular subject sometimes “attract” the agreement with the verb, despite not being grammatically licensed to do so. This phenomenon generates agreement errors in production and an increased tendency to fail to notice such errors in comprehension, thereby providing a window into the representation of grammatical number in working memory during sentence processing. Research in this topic, however, is primarily done in related languages with similar agreement systems. In order to increase the cross-linguistic coverage of the processing of agreement, we conducted a self-paced reading study in Modern Standard Arabic. We report robust agreement attraction errors in relative clauses, a configuration not particularly conducive to the generation of such errors for all possible lexicalizations. In particular, we examined the speed with which readers retrieve a subject controller for both grammatical and ungrammatical agreeing verbs in sentences where verbs are preceded by two NPs, one of which is a local non-subject NP that can act as a distractor for the successful resolution of subject-verb agreement. Our results suggest that the frequency of errors is modulated by the kind of plural formation strategy used on the attractor noun: nouns which form plurals by suffixation condition high rates of attraction, whereas nouns which form their plurals by internal vowel change (ablaut) generate lower rates of errors and reading-time attraction effects of smaller magnitudes. Furthermore, we show some evidence that these agreement attraction effects are mostly contained in the right tail of reaction time distributions. We also present modeling data in the ACT-R framework which supports a view of these ablauting patterns wherein they are differentially specified for number and evaluate the consequences of possible representations for theories of grammar and parsing.

## 1. Introduction

A fundamental feature of language comprehension in real time is the online integration of grammatical information in the form of structural cues expressed morphologically on individual lexical items. For instance, many languages display grammatical agreement—a process whereby verbs co-vary in form with features of their arguments. Integrating agreement cues to resolve verb-argument agreement dependencies provides the parser with valuable information concerning structural relations in the input and therefore provides important clues to the correct parse. Moreover, humans are quite good at completing this resolution: it is conducted relatively quickly, and failures to resolve agreement dependencies result in failures of parsing in many instances.

Despite this relative aptitude in comprehending agreement, speakers do make mistakes in both the comprehension and production of agreement dependencies. Since the initial study of Bock and Miller (1991), a large amount of theorizing concerning the nature of this integration has been based upon failures of agreement called *agreement attraction* errors. In an agreement attraction error, an agreeing element does not correctly match its controller in all features but instead matches a local *distractor* or *attractor* in a subset of the mismatching features. A key property of these errors is that this distractor NP is typically thought to be grammatically inaccessible insofar as it is not normally capable of controlling agreement because of its structural position. For instance, in English subject-verb agreement dependencies, attraction errors have been noted for several configurations, including prepositional phrase modifiers/complements, relative clauses, and the like (1)^1^:

(1) a. *The sheer weight* of **all these figures** ***make*** them hard to understand.
      (based upon Ronald Reagan 13 October 1982; quoted in Francis, 1986 and Wagers et al., 2009)
      b. *The boy* that liked **the snakes** ***sleep*** throughout the afternoon.
      (based upon Bock and Miller, 1991)
      c. *The request* to begin **the projects** ***were*** overwhelming because of the cost.
      Tucker and Wagers (2010)
      d. *Studying* **micro-climates** like this ***have*** helped me to understand if it's gonna be a minor event, or a catastrophic one.
      (Jim Wood, professional blog post 13 August 2012^2^)

Errors such as these are often discussed by both grammarians and syntactic theorists alike (see Jespersen, 1924; Zandvoort, 1961; Kimball and Aissen, 1971; Quirk et al., 1985; Francis, 1986; Kayne, 2000; Den Dikken, 2001; *inter alia*) and—despite their *prima facie* ungrammaticality—are common in both everyday speech and formal writing. Both production and comprehension studies have shown that the probability of agreement attraction errors is influenced by a large number of factors, including linear order, relative structural embedding, and the amount of featural overlap between distractor and verb (Bock and Cutting, 1992; Bock and Eberhard, 1993; Vigliocco and Nicol, 1998; Pearlmutter et al., 1999; Hartsuiker et al., 2001; Franck et al., 2002; Thornton and MacDonald, 2003; Haskell and MacDonald, 2005; Wagers et al., 2009; *inter alia*).

The factors which have been shown to influence the possibility of these errors include both processing and grammatical constraints. For instance, several researchers beginning with Bock and Miller (1991) have noted that agreement attraction errors are asymmetric in both their occurrence and salience in English. Specifically, whereas errors leading to erroneously plural verbs (2a) are commonly produced and more difficult to notice, erroneously singular verbs (2b) are rarely produced and seem much more salient to speakers:

(2) a. *The key* to **the cabinets** ***have*** become rusty from years of disuse.       (**SG** → **PL**)
      b. *The keys* to **the cabinet** ***has*** become rusty from years of disuse.         (**PL** → **SG**)

One plausible explanation for this asymmetry is the grammatical notion of markedness, wherein one marked value of a feature (in this case, plural) is defined by its opposition to another unmarked value (in this case, singular). By tapping into this grammatical notion, the reason for the particular direction of this asymmetry becomes explainable as attraction to the marked plural case in (2a). In (2b), on the other hand, the presence of an unmarked attractor means the verb is less easily misconstrued.

Similarly, the grammatical notion of a syntactic hierarchy has also been shown to be relevant by both Bock and Cutting (1992) and Franck et al. (2002), among others. In the Franck et al. (2002) study, the authors contrasted preambles such as (3) in a sentence production study to determine whether linear distance or syntactic prominence (defined in terms of structural height in a parse tree) contributes more to attraction. In these preambles, the linearly closest NP is not the structurally most prominent NP computed in terms of structural height:

(3) a. *L'-ordinateur* avec **le programme des expériences** …
          the-computer with the program of.the experiments …
          “*The computer* with **the program** of **the experiments** …”

    b. *L'-ordinateur* avec **les programmes** de **l'expérience** …
          the-computer with the programs of.the experiment …
          “*The computer* with **the programs** of **the experiment**…”

In (3), the PP containing *expérience(s)* is a complement, and therefore structurally contained within the NP headed by *programme(s)*. The authors observed that the syntactically higher noun [*le(s) programme(s)*] has a larger impact on attraction error rates than the linearly closest noun (*des expériences*) leading the authors to conclude that syntactic hierarchical prominence plays a larger role than linear adjacency in modulating attraction rates.

On the other hand, processing constraints clearly matter, as well. Most concretely, attractions are errors, and only appear in a subset of observations for any given language community^3^. Moreover, emerging comprehension literature has shown that attraction errors in comprehension only occur in *ungrammatical* utterances, not grammatical ones (see, e.g., Wagers et al., 2009; Tucker and Wagers, 2010; Tanner et al., 2014). Thus, one does not find the comprehension correlates of attraction in examples such as (4):

(4) *The key* to **the cabinets** ***has*** become rusty from years of disuse.

Despite the fact that the attractor noun phrase mismatches the subject and is plural, reading times at *has* and error rates in speeded grammaticality studies do not reflect difficulty for the parser. Thus, error rates on examples such as (4) are low, and reading times at *has* do not differ from normal reading times for grammatical verbs. The explanation given for this asymmetry by Wagers et al. (2009) is that the attraction in ungrammatical sentences is the result of the parser's attempt to interpret an obviously erroneous verb by searching working memory for a matching noun phrase. Crucially, Wagers and colleagues contrast this with a view wherein grammatical representations are themselves fallible by observing such a view should apply equally in grammatical and ungrammatical utterances. This parsing strategy therefore provides a superior explanation for the grammaticality asymmetry in attraction than a view more wedded to grammatical representation.

### 1.1. Representations and processes

A recently emerging hypothesis concerning the proper interpretation of dependency errors takes them to be a failure of the working memory implementation of agreement dependencies (Badecker and Kuminiak, 2007; Badecker and Lewis, 2007; Wagers et al., 2009; Dillon et al., 2013) following a general hypothesis that at least some of the processes involved in language comprehension are underwritten by a kind of skilled memory retrieval (Lewis and Vasishth, 2005; Lewis et al., 2006). Architecturally and programmatically, viewing dependency resolution as skilled memory retrieval allows the development of explicit hypotheses about the relationship between behavioral results and architectural claims about language comprehension insofar as researchers are forced to be explicit about both representational and procedural commitments.

In comprehension-as-retrieval models, some or all agreement morphology on a lexical item triggers a working memory retrieval event wherein the system attempts to find an available controller in a content-addressable memory. In order to do so, a procedural component searches all available chunks (constituents) in memory in parallel and attempts to locate a match along several cue dimensions, with the winner being decided by which element matches along the most dimensions. When the controller matches the agreeing element in all grammatical features, the number of matching retrieval cues will result in a proper retrieval of the true grammatical controller. However, when the controller and agreeing element do not match in all cues, those mismatched cues which the distractor bears can, in some instances, be sufficient to trigger an erroneous retrieval of the distractor, resulting in an attraction error.

The retrieval hypothesis is well-suited to explain the sensitivity of attraction to mismatches in controller and distractor cues (Bock and Miller, 1991; *et seq*.), the absence of attraction-like illusions of ungrammaticality in grammatical utterances (Wagers et al., 2009), and the relative error proportions in various constructions (Dillon et al., 2013). Finally, recently emerging work suggests that memory models are also, when combined with proper representational specifications, well-suited to explaining differing behavioral profiles for at least some different kinds of grammatical dependencies (Dillon et al., 2013; though see Parker, 2014 for some critical discussion).

Linking agreement attraction errors to more general comprehension models provides for some important possibilities for research into both the grammar and parsing. Specifically, as Dillon et al. (2013) demonstrate, in places where experimental data are suggestive of a particular representational commitment in the parser, modeling can provide additional evidence for this commitment when it dovetails with experimental results. Moreover, the success of memory models in accounting for particular experimental results across a range of languages adds to the validity of the models themselves. In order to do this modeling of experimental results, however, researchers must stake particular claims about the relationship between parsing and grammar in order to decide on representations and processes in the models. Any such claims, therefore, help elucidate the connection between parsing *per se*, grammar, and working memory.

By contrast to the memory models, several alternatives have been proposed which view agreement attraction as either grammaticalized alternatives (Kimball and Aissen, 1971; Kayne, 2000; Den Dikken, 2001) or an improper representation driven by feature movement or percolation of number features to incorrect nodes in syntactic trees (Nicol et al., 1997; Vigliocco and Nicol, 1998; Franck et al., 2002; Eberhard et al., 2005). However, as was first pointed out by Wagers et al. (2009), models which eschew the role of memory are only successful insofar as one can identify correlates of their representational claims in all aspects of processing behavior. Grammaticalization models assume that, at the very least, attraction should be possible outside of error contexts, a finding which has yet to be conclusively demonstrated. As for representational models, memory models have been argued to be superior to purely representational approaches in understanding the comprehension of grammatical sentences which contain the structural configurations supporting the creation of erroneous representations. As Wagers et al. (2009) have argued, erroneous representations should be possible in ultimately grammatical utterances, yet experiments designed to test for the presence of “agreement attraction” in grammatical utterances consistently yield null results (see Wagers et al., 2009; Tanner et al., 2014; and our results below). We therefore conclude, with these authors, that memory models provide a better avenue for exploration in the service of explaining possibly erroneous dependency processing in natural language and couch the study reported here in memory retrieval terms.

### 1.2. Crosslinguistic considerations

What working-memory models require, however, is a well-understood theory of the relationship between formal linguistic features usually referenced in linguistic theories of agreement (such as those proposed by Chomsky, 1995, 2000, 2001; and related work) and the cues used in models of working memory tasks. It is therefore conspicuous that the prevailing views on feature-cue mapping have been developed with a comparatively small sample of languages in mind: the majority of studies have examined either Germanic languages such as Dutch, English, and German or Romance languages such as French, Spanish, and Italian (For English, see any of the previously cited works except Franck et al., 2002, among many others. For Dutch, see Hartsuiker et al., 1999; Meyer and Bock, 1999; Bock et al., 2001; Kaan, 2002; Hartsuiker et al., 2003; for German, see Hartsuiker et al., 2003; Häussler, 2009; for Spanish, see Vigliocco et al., 1996; Antón-Méndez et al., 2002; Franck et al., 2008; Lago et al., 2014; for French, see Fayol et al., 1994; Vigliocco et al., 1995; Vigliocco and Franck, 2001; Franck et al., 2002, 2008, 2010 and for Italian, see Vigliocco et al., 1995; Vigliocco and Franck, 1999, 2001; Franck et al., 2006, 2008). The only exceptions to this tendency involve two studies on the Slavic languages Russian (Lorimor et al., 2008) and Slovak (Badecker and Kuminiak, 2007), however even this sample of languages is wholly contained within the larger Indo-European family. To our knowledge, no studies of agreement attraction exist in languages outside Indo-European. A theory of the relationships connecting grammar, parsing, and working memory is ultimately a theory about the implementation of language in the mind, and therefore would benefit from the largest possible cross-linguistic coverage since it is conceivable that there is crosslinguistic variation here.

This lacuna is additionally striking when one considers the possible range of variation in the expression of verbal agreement. Germanic and Romance languages display subject-verb agreement for grammatical number, and while nominals in these languages have formal gender, this gender does not impact the subject-verb agreement system. This is not true of the Slavic languages studied by Badecker and Kuminiak (2007) and Lorimor et al. (2008), where converging evidence seems to suggest that gender does play a role in attraction. However, in these languages, nominal morphology also includes grammatical case-marking, which is shown to play a confounding role insofar as the case a nominal bears helps to disambiguate its grammatical function (for similar evidence in German, see Häussler, 2009). In these languages, it may be possible to set up an attraction configuration involving gender, but grammatical case on the attractor serves to disambiguate its grammatical role in a way which drives down attraction rates. It is thus important to broaden the empirical base of agreement attraction errors by considering their properties in languages outside the handful of well-studied languages in this domain of research, as the restriction to these languages could in principle unduly influence representational commitments made on the basis of particular kinds of verbal agreement paradigms.

A crosslinguistic perspective is an important one for addressing a pressing question in memory models concerning the distinction between a grammatical feature and a processing/memory retrieval cue. While it is clear that theoretical work can identify features utilized by the grammatical system, it is an open question how these features map onto cues which are used in the memory retrieval system. Just because grammar provides a feature as part of a contrast does not mean that the parser must utilize this feature in dependency resolution. Here, again, the memory retrieval models force an explicit commitment insofar as predictions about which constituents in memory are retrieved (as well as the latency of that retrieval) can only be made when one is explicit about the inventory of cues available to the system. Investigating these questions in languages which utilize different grammatical features in distinct ways is therefore a necessary part of understanding the feature-to-cue mapping.

Finally, an additional reason that crosslinguistic consideration is important relates to the way that memory models relate available cues to available activation in the system. Since the eventual retrieval target is the chunk in memory which has the highest activation at the retrieval event, and this activation is itself a function of two things: (1) the number of cues which a chunk shares with the goal and (2) the total number of chunks associated with each individual cue. A corollary of this architecture is that the number of available cues in a language directly modulates the amount of activation in the system. Adding more morphological features to discriminate NPs in memory should, in principle, drive down error rates. It is therefore an open question whether one expects agreement attraction in a language which is sufficiently morphologically rich in its verbal agreement^4^. Understanding the predictions such a system makes as available cues vary crosslinguistically is therefore an important way of validating such architectures more generally. Here, again, we believe testing memory models across the widest variety of languages should be an important research objective.

### 1.3. The relevance of arabic

It is here where Modern Standard Arabic (MSA; also equivalently just “Arabic” in what follows) is particularly well-suited as a language of interest. Arabic is spoken by over 200 million people worldwide and MSA is a *lingua franca* used in writing and formal speech across different regional varieties of spoken Arabic (as well as within-dialect groups). MSA is relevant for agreement attraction studies because it has verbal agreement for grammatical gender for both masculine and feminine subjects, a dual number (Ryding, 2005, pp. 438–444), and case marking which is optional on NPs under particular circumstances (Ryding, 2005, pp. 165–205). These kinds of agreement are in addition to the more standard singular/plural distinction seen in languages such as English and demonstrated for Arabic in (5)^5^:

(5) a. 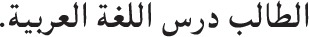          at^ˤ^-t^ˤ^ aalib        daras-a
          the-student(.MASC) study-3.MASC.SG.PERF
          al-luɣa    al-ʕarabiyya.
          the-language the-arabic
          “The student studied Arabic.”

    b. 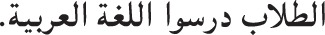          at^ˤ^-t^ˤ^ulaab        daras-uu
          the-student(.MASC.PL) study-3.MASC.PL.PERF
          al-luɣa    al-ʕarabiyya.
          the-language the-arabic
          “The students studied Arabic.”

Additionally, MSA has two distinct strategies for forming plurals on nouns: (1) a plural formed by suffixation, called the “sound” plural (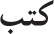/*t*^ˤ^aaliba—t^ˤ^aalib-aat, “student ~ students (fem.)”) and (2) a plural formed by ablaut, called the “broken” plural in traditional Arabic grammar (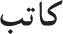
∫ajx ~ ∫ujuux, “sheikh ~ sheikhs”). While the latter strategies for pluralization would normally be referred to as “irregular” in the English literature, the broken/ablauting plural strategy is very common in Arabic—if not more common than the sound/suffixing plural strategy (see, e.g., Ryding, 2005, pp. 132–204). For nouns which take suffixes in the plural, these suffixes are absolutely regular: in the feminine there is only /-aat/ (Ryding, 2005, pp. 132–133). For masculine nouns which take suffixing plurals, there are up to two suffixes, /-uun/ for nominative case and /-iin/ for genitive and accusative case (Ryding, 2005, p. 140)^6^. By contrast, the number of broken plural patterns is considerably higher: (Ryding, 2005) lists 26 distinct patterns and (McCarthy and Prince, 1990b), following (Wright, 1889a,b), give 31 patterns. This sound/broken contrast is an important one because it cross-cuts other grammatical concerns in Arabic: what type of case morphology is available for a noun depends on what kind of plural it takes (Ryding, 2005, pp. 165–204); affects theoretical conceptions of morphological process (McCarthy and Prince, 1990b); and may affect lexical access at the word level (Mimouni et al., 1998).

These two types of plurals are of particular interest because they allow investigation of the representation of plurality in both linguistic representation and the working memory system. A recurring question in experimental work on Semitic is to what extent grammatical theories concerning word representation postulate representational constructs which are useful for psycholinguistic theorizing. Specifically, traditional Arabic grammars characterize most words as consisting of a consonantal root (made up of two to five consonants) interleaved among vowels in a so-called prosodic template (see, e.g., Ryding, 2005, pp. 45–50), a characterization which has heavily influenced linguistic theories of the language, as well (see, for example, McCarthy, 1979, 1981; McCarthy and Prince, 1990a,b; Ussishkin, 2000, 2005; Tucker, 2010, 2011; Ussishkin et al., 2015). For instance, (6) gives examples of several distinct words all sharing the root $\sqrt{\text{ktb}}$:

(6) a. 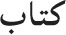/kataba, “he wrote”
    b. 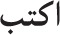/kaataba, “he corresponded”
    c. 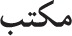/kitaab, “a book”
    d. 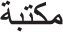/uktub, “write!”
    e. 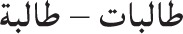/maktab, “an office/desk”
    f. 
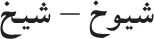
/maktaba, “a library”

Formal Arabic grammar is mostly uniform in its description of Arabic morphology in these root and template terms. However, depending on the part of grammar being considered, psycholinguistic work has found variable evidence for the template, mainly from priming (for Hebrew, see Frost et al., 1997; Deutsch et al., 1998; Frost et al., 2000; for Maltese, see Ussishkin and Twist, 2007; Ussishkin et al., 2011; and for Arabic, see Boudelaa and Marslen-Wilson, 2001, 2004b,a, 2005; Boudelaa et al., 2010; Boudelaa and Marslen-Wilson, 2011). Notably, this reliance on priming has led to most conclusions about the psycholinguistic validity of these representations being confined to lexical decision independent of sentential context. However, one thing which is not addressed in most of the recent work on Semitic morphosyntax is how this root-and-pattern system interacts with the representation of plurality in both grammar and parsing (a notable exception being the early work of McCarthy, 1981, where it is explicitly claimed that templates are morphemes which bear grammatical content). For instance, one can easily wonder, for broken plurals, where the grammatical plural feature is located in the representation and how such a representation translates into use for parsing. Given that there is enough linguistic and psycholinguistic evidence that suggests one should take the broken/ablauting vs. sound/suffixing contrast seriously on Arabic-internal terms, here we attempt to see whether this contrast is informative for diagnosing how the processing system encodes nouns in general and plurality more specifically. While this question is particularly salient for Semitic-internal debates, it is germane to research on morphological representations outside of this language family, as well insofar as other languages have similar representations for morphological features.

### 1.4 The present study

As promising as the grammatical situation is in MSA for probing the mapping between features and cues in agreement dependency resolution, it remains to be seen whether or not agreement attraction exists for the standard number features seen in previous studies. The study reported here took up this question by considering the resolution of agreement dependencies involving plural attractors. In better-studied languages such as English, one finds that plural attractors occasionally condition erroneous plural verbal morphology, as in (7):

(7) *The key* to **the cabinets** ***are*** rusty from years of disuse.

In production, agreement attraction errors manifest as production of the erroneous verb (Bock and Miller, 1991; *et seq*.), whereas in comprehension attraction errors manifest as facilitation on ungrammatical verbs when attraction configurations are present (Pearlmutter et al., 1999; Wagers et al., 2009; Dillon et al., 2013; Tanner et al., 2014) or as a reduced-amplitude P600 in attraction configurations in ERP research (Tanner et al., 2014).

The study reported below therefore also investigated the representation of number cues across different kinds of plurals in Arabic using self-paced reading while counterbalancing attractor plural type. We predicted the existence of such errors in comprehension in MSA as a facilitation to erroneously plural verbs in the presence of a plural attractor relative to singular attractors in the same context. As for plural type, we were more reserved in our prediction, being unsure as to the theoretical status of plural types in the language. It has previously been observed for English (Bock and Eberhard, 1993) that irregular plural formation (*ox*~*oxen*, *mouse*~*mice*) on an attractor NP does not condition differential error rates. However, in that production study, the focus was on a language for which ablauting plurals are exceptionally rare and form a small corner of the nominal inventory of the language. In MSA, the relative abundance of ablauting plurals may very well mean differential behavior between suffixing sound plurals and ablauting broken plurals. Any such difference, in turn, would have implications for the mapping between grammatical plural features and plural retrieval cues on NP constituents in working memory.

## 2. Self-paced reading

As a prerequisite for any systematic investigation of the unique properties of Arabic morphology and their effect on agreement attraction, it is first necessary to be sure that attraction errors of the kind documented for other languages occurs in MSA. We think this an especially important contribution given the relative inhospitability of the Arabic agreement system to agreement attraction errors: the system involves a large number of cues (person, number, and gender) which assist the parser in retrieving the correct subject. In order to determine whether attraction errors are possible in MSA, an experiment was designed based upon the relative clause stimuli in the initial (Bock and Miller, 1991) study. The purpose of this experiment was to ensure that subject-verb agreement errors for singular and plural number do occur in a relatively frequently-occurring grammatical configuration that allows for subsequent manipulation of less well-studied number and gender alternations.

We therefore test the Arabic equivalents of a subset of preambles from the Bock and Miller (1991) study on English. Specifically, Bock and Miller (1991) tested production agreement errors elicited after giving participants preambles such as *The boy(s) that liked the snake(s) …* which varied based on the number for the subject [*the boy(s)*] and the local distractor noun [*the snake(s)*]. However, we were also interested in the real-time processing properties of attraction errors, so we investigate comprehension by measuring the reading times for complete versions of these sentences. This allowed us to simultaneously remain close to the original phenomenon in English while simultaneously exploring the comprehension of agreement in Arabic.

### 2.1. Method

#### 2.1.1. Participants

Participants were 114 native speakers of Arabic from the University of the United Arab Emirates and NYU Abu Dhabi student bodies (113 female; mean age 21.1 years)^7^. All participants had no history of language disorders and read MSA regularly. Each participant provided written informed consent and was compensated for their participation. This experiment was approved by the NYU Abu Dhabi Institutional Review Board and the UAEU Ethics Committee.

#### 2.1.2. Materials

A set of 48 sentences was constructed, each containing a subject relative clause with an animate object modifying the animate subject of a transitive verb. Subject relative clauses were chosen because they are a long-standing example of a configuration which creates agreement attraction errors (e.g., Bock and Miller, 1991) and are relatively common in MSA. In this sense they are a better choice than the canonical *NP—PP* configuration in more memorable examples such as *The key to the cabinets*…. The issue these constructions pose for the present study is that Arabic does not easily allow adverbs to be placed between subject and verb (Tucker, 2011) the inclusion of which was a *desideratum* of our stimuli. This is because, following (Wagers et al., 2009), we wished to insert an adverb or adverbial prepositional phrase between the end of the relative clause and the target main clause verb in order to mitigate plural NP spillover effects into the target region. All the stimuli therefore had the structure *NP1—Complementizer—RC Verb—NP2—Adv/PP—Verb—Continuation*. An example of such a sentence appears in (8):

(8) 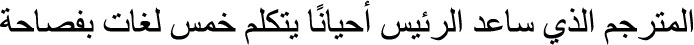 
     ʔal-mutarʒim-u      ʔalla ii
       the-translator-NOM COMP.MASC.SG
       saaʕad-a       ʔal-raʔiis-a        ʔaħjaanan
       helped-3.MASG.SG the-president-ACC often
       ja-takallamu         xamsata luɣaat-in
       3.SG.MASC-speaks five    languages-ACC
       bi-fas^ˤ^aaħatin.
       with-fluency
 
     “The translator who helped the president often speaks five languages fluently.”

Several constraints guided the construction of these experimental sentences: Firstly, Arabic has a series of prepositions which are only a single syllable/orthographic character and which are written with no space separating them from the complement NP. Only these prepositions were used in constructing adverbial PPs, meaning that the buffer region between distractor NP and target verb was no more than one orthographic word for any sentence. Secondly, for any given sentence both the subject and distractor NP were the same grammatical gender (masculine or feminine), and the total number of masculine and feminine gender nouns was balanced across sentences (24 masculine, 24 feminine). We decided not to allow different genders in the same sentence because of the confound introduced by the complementizer in MSA, as it must agree with definite head nouns (Ryding, 2005, pp. 322–324). Because of this, the true subject would receive an additional disambiguating cue from the complementizer's gender. However, the complementizer does inflect for grammatical number, meaning that in our stimuli the true subject receives reinforcement from the singular complementizer in conditions with plural attractors.

Additionally, we sought to vary the kind of plural which the attractor NP takes in the plural conditions. However, grammatical case in Arabic is normally optionally expressed in diacritics which are not written in everyday MSA, with the exception of suffixing masculine plurals, which do show an orthographic distinction between accusative and nominative case (represented by a change in an orthographically obligatory long vowel). In order to avoid adding a potentially disambiguating cue, case-marking, all masculine distractor NPs took broken plurals and all feminine distractor NPs took suffixing plurals. We also opted to conflate gender and plural type because MSA does not furnish a sufficiently large number of broken feminine plurals which refer to animates. This strategy allowed balancing of gender and suffixation in the plural in a grammatically natural way without introducing confounds from orthographically-represented grammatical case. This design allows us to check whether different pluralization processes (ablaut vs. suffixation) influence agreement attraction effects differently, although in our design this is necessarily confounded with grammatical gender.

In addition to gender and plural type, the sentences were also counterbalanced for whether the target verb appeared in the present or past tense. This was done because MSA has two distinct series of affixes for verbal agreement: (1) the present tense, with both a prefix and suffix and (2) the past tense, with suffixes only (see, e.g., Ryding, 2005, pp. 438–444). Counterbalancing in this way allowed conclusions to be drawn about agreement independent of the specific affix series employed. We did assess the effect of tense/aspect in the reading time results presented below and found no effect of the affix series employed.

Finally, stimuli in Arabic must stake a position on the orthographic representation of short vowels. Arabic is written in an alphabet which only represents long vowels, where short vowels are only written in religious texts, poetry, and texts for language-learners. In everyday formal written Arabic, short vowels are sometimes employed when an orthographic string is lexically ambiguous without some short vowel specification or in a way which is not resolvable from sentential context. The effects of adding superfluous or normally unwritten short vowels to Arabic language stimuli is understudied, and therefore a point of particular concern. In our stimuli, we therefore employed minimal diacritics only where lexical ambiguity would result if the diacritics were not used. This is a common scheme for representing diacritic marks in MSA and matches what is seen in everyday formal writing in the Arab world.

For each experimental sentence, four variants were constructed by systematically varying the morphological number of the object of the relative clause (*NP2*, the *attractor* or *distractor*) and the main clause verb (the *Verb*). This resulted in four conditions per sentence which are labeled according to the number of *NP2* and *Verb*: (S)ingular or (P)lural. We call the conditions in which the verb is plural *ungrammatical* conditions, since all subjects were singular in the experimental items. A complete item set appears in Table 1 and the complete list of sentences appears in the Supplementary Materials.

**Table 1:** **A complete item set for one sentence for the experiment**.

**Condition**	**NP1**	**Comp**	**RCV**	**NP2**	**Adv**	**V**	**Continuation**
	**R1**	**R2**	**R3**	**R4**	**R5**	**R6**	**R7–R10**
Sg/Gram	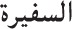	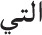	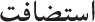	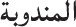	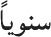	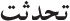	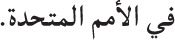
	The ambassador	who	hosted	the diplomat	yearly	spoke(FEM.SG)	at the United Nations
Sg/Ungram	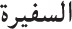	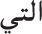	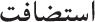	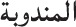	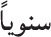	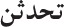	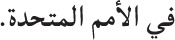
	The ambassador	who	hosted	the diplomat	yearly	spoke(FEM.PL)	at the United Nations
Pl/Gram	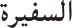	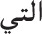	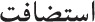	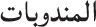	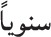	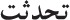	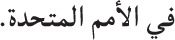
	The ambassador	who	hosted	the diplomats	yearly	spoke(FEM.SG)	at the United Nations
Pl/Ungram	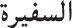	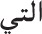	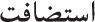	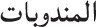	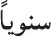	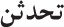	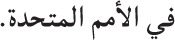
	The ambassador	who	hosted	the diplomats	yearly	spoke(FEM.PL)	at the United Nations

The 48 sets of four sentences were distributed across four lists in a Latin Square design and combined with 144 grammatical filler items of a similar length in order to distract from the target items. None of the fillers contained the subject relative clause construction contained in the stimuli. This resulted in a filler-to-item ratio of 3:1 with 25% of the sentences being ungrammatical.

In this study we expect several things based upon the previously published studies for Germanic, Romance, and Slavic languages. Specifically, we expect to find a main effect of grammaticality in the critical verb region (RCV) and subsequent regions owing to possible spillover. Moreover, we expect to find a interaction between this factor and the attractor number factor in the critical verb region (possibly including spillovers) driven by slower reading times for the Sg/Ungram condition relative to the Pl/Ungram condition—this is the attraction configuration. Moreover, we expect to find no difference between the two grammatical conditions, Sg/Gram and Pl/Gram, given that no comprehension attraction effects have been observed in the previous literature. Additionally, following the discussion in Wagers et al. (2009), we expect to find a main effect of attractor number alone in the attractor region (NP2), a plural reading time effect noted in that work but not presently well-understood. Finally, we have no *a priori* expectations about the nature of the effect of plural type, but suspect that it is relevant for on-line processing given its centrality in the grammatical and lexical access literature.

#### 2.1.3. Procedure

Subjects were seated comfortably up to eight at a time at a table in a quiet room in front of computers on which the experimental software had been pre-loaded. Sentences were presented using the Linger software (Rhode, 2003) in a self-paced word-by-word moving window paradigm (Just et al., 1982). Each trial begin with the display of a screen containing the sentence masked by dashes (including spaces and punctuation). Each time the participant pressed the space bar, a single word was revealed and the previous word re-masked. All items were presented in the Courier New Arabic font in 28pt bold type. A yes/no comprehension question (not an acceptability judgment) followed each sentence, appearing on the screen all at once. Comprehension questions were designed in such a way that the answer could be provided independent of experimental manipulations—no questions asked about the attractor NP or the main clause verb. None of our comprehension questions required lexical elaboration of the item or difficult semantic processing. A majority of the comprehension questions asked about the relative clause verb or the post-critical region continuation. As an example, the item *The student who saw the professor(s) yesterday studied electrical engineering at the university*. was followed by the question *Did the student see someone?*. The ‘f/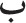
’ key was used for “yes (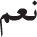)” and the ‘j/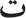
’ key used for “no (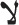).” Onscreen feedback was provided for both correct and incorrect answers. Participants were instructed to read at a natural pace ensuring comprehension and were not alerted to the presence of grammatical errors in the stimuli. The order of sentence presentation within each list was randomized by the experimental software for each participant. Four practice items were presented before the start of the experiment.

#### 2.1.4. Data analysis

Subjects which were less than 70% accurate on comprehension questions were excluded from further analysis on the grounds that they were not sufficiently attentive to the task; this criterion resulted in the exclusion of 10 subjects. Outliers were handled by Winsorizing the extreme 5% of the data (Ratcliff, 1993). No other exclusion criteria were used.

Data from both the comprehension question responses and remaining region-by-region reaction times were analyzed using mixed effects regression (Baayen et al., 2008). The answers to the comprehension questions were entered into several logistic mixed effects models including experiment, condition, and experimental independent variables (attractor number and grammaticality) as fixed effects and subjects and items as random effects with intercepts only. Self-paced reading data for each region of interest (R4, the attractor region, through R8, the second post-critical verb region) were entered into a linear mixed effects model fit using restricted maximum likelihood estimation with both subjects and items as random effects and several predictors as fixed effects: (1) attractor number, (2) grammaticality, (3) attractor plural type (ablauting/suffixing), (4) item order in the experimental presentation, (5) log frequency of the plural of the attractor according to the arabiCorpus (Parkinson, 2012), (6) word length in characters, (7) the previous region's reading time, and (8) interactions of terms (1–3). Categorical predictors were dummy-coded using the following default values: (1) grammaticality = grammatical, (2) attractor number = singular, and (3) gender/plural type = feminine (sound/suffixing) and neither categorical nor continuous predictors were centered. Our random effects structure was comprised of intercepts for subjects and items. For both the comprehension and reading-time results we used a minimal random effects structure in order to ensure convergence of the models (but see Barr et al., 2013). Degrees of freedom were estimated using the Welch-Satterthwaite approximation in order to calculate a *p*-value; we therefore report *t*-values directly instead of *z*−scores or 95% confidence intervals generated by bootstrapping or MCMC sampling. More details on the modeling for the reading time results can be found in the Supplementary Materials.

### 2.2. Results

#### 2.2.1. Comprehension question accuracy

The mean comprehension question accuracy pooled across subjects and items to both experimental items and fillers was 88.2% and was significantly lower for experimental items (80.0%) than for fillers (91.1%) (logistic mixed-effects model $\hat{\beta }$_1_ = 1.44; *z* = 19.80; *p* < 0.0001). We believe this lower accuracy to the experimental item comprehension questions is due to errors in the construction of some of the questions themselves. Participants reported confusion over the intent of seven of the questions; with these questions excluded, experimental item accuracy increased to 86.1%. Nevertheless, we exclude data from these items when the comprehension question was answered incorrectly in the reading-time analysis which follows, as this is the most conservative approach.

Accuracy rates for singular attractors were 81.0 ± 1.2% (with standard errors computed over participant means) for grammatical sentences and 78.8 ± 1.3% for ungrammatical sentences. For plural attractors, accuracy rates were 82.7 ± 1.3% for grammatical sentences and 76.0 ± 1.4% for ungrammatical sentences. The configuration of plural attractor and grammatical verb had a significant impact on question accuracy ($\hat{\beta }$ = 0.36; *z* = 2.11; *p* = 0.03) such that participants were more likely to be correct in this condition relative to the attraction configuration of plural attractor and ungrammatical verb.

#### 2.2.2. Self-paced reading

The self-paced reading results for all items are presented immediately below. Because of our *a priori* interest in the impact of grammatical and lexical access-related differences in plural formation type on agreement attraction, we provide some additional results by gender/plural type, as well. In what follows, we focus our reporting on the results of the experimental manipulations of Attractor Number, Grammaticality, and Gender/Plural Type. We do not comment on the presence of effects due to the frequency of the attractor, word length, or previous region's reading time, as these predictors are commonly found to be explanatory in reading time studies and we have nothing to add here to their interpretation as determinants of reading time.

#### 2.2.2.1. All items

**Figure 1:**
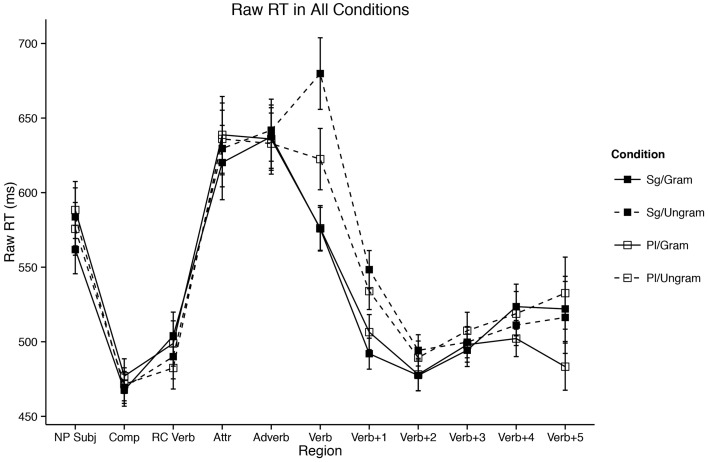
**Self-paced reading results**. Region by region means segregated by attractor number and verb number. Error bars represent the standard error of the mean computed across subject averages.

The results from the experiment are presented in Figure 1 and the mixed-effects model results for the attractor region (R4) and critical verb region (R6) appear in Tables 2, 3. Linear mixed-effects model results for all other regions of interest are included in the Supplementary Materials.

**Table 2:** **Table of coefficients for a linear mixed effects regression with gender/plural type for the attractor region (R4)**.

**Factor**	**$\hat{\beta }$**	***t***	***df***	***p***
**Intercept**	**382.05**	**6.01**	**100.00**	**<0.0001**
Attr	14.47	0.65	757.00	0.51
Grammaticality	16.94	0.92	3926.00	0.36
**Gender/Plural Type**	**72.00**	**2.53**	**143.00**	**0.01**
**Item Order**	**−1.29**	**−15.18**	**3964.00**	**<0.0001**
Attr Frequency	−14.39	−1.50	104.00	0.14
**Length**	**35.62**	**4.71**	**72.00**	**<0.0001**
**Previous Region RT**	**0.17**	**11.99**	**4036.00**	**<0.0001**
Attr × Gram	−27.82	−1.07	3927.00	0.28
**Attr × Gender**	**−62.24**	**−2.37**	**3833.00**	**0.02**
Gram × Gender	−6.65	−0.26	3929.00	0.80
Attr × Gram × Gender	33.71	0.92	3931.00	0.36

*p-values computed using the Welch-Satterthwaite approximation. Predictors significantly different from 0 at α = 0.05 highlighted in bold*.

**Table 3:** **Table of coefficients for a linear mixed effects regression with gender/plural type for the critical verb region (R6)**.

**Factor**	**$\hat{\beta }$**	***t***	***df***	***p***
**Intercept**	**557.54**	**13.61**	**122.00**	**<0.0001**
Attr	24.39	1.44	1639.00	0.15
**Grammaticality**	**102.56**	**6.70**	**3657.00**	**<0.0001**
Gender/Plural Type	−11.90	−0.53	152.00	0.60
**Item Order**	**−1.38**	**−19.90**	**3961.00**	**<0.0001**
Attr Frequency	16.24	1.90	129.00	0.06
**Length**	**28.68**	**3.72**	**73.00**	**0.0004**
**Previous Region RT**	**0.04**	**4.81**	**4062.00**	**<0.0001**
**Attr × Gram**	**−72.40**	**−3.44**	**3925.00**	**0.0006**
Attr × Gender	−14.50	−0.68	3927.00	0.49
**Gram × Gender**	**−59.85**	**−2.50**	**1157.00**	**0.01**
Attr × Gram × Gender	39.08	1.31	3928.00	0.19

*p-values computed using the Welch-Satterthwaite approximation. Predictors significantly different from 0 at α = 0.05 highlighted in bold*.

The relative clause attractor region (R4) contained a main effect gender/plural type such that masculine attractor NPs were read more slowly than feminine attractor NPs [$\hat{\beta }$ = 72.00; *t*_(143.00)_ = 2.53; *p* = 0.01]. Additionally, there was an interaction between gender/plural Type and attractor number [$\hat{\beta }$ = −62.24; *t*_(3833.00)_ = −2.37; 0.02] which was driven by significantly longer reading times to plural attractors for feminine attractors [*t*_(207)_ = 2.99; *p* = 0.003; plural mean = 674.80 ms; singular mean = 629.10 ms]. The same was not true of masculine attractors [*t*_(207)_ = −1.11; *p* = 0.27; plural mean = 600.42 ms; singular mean = 615.34 ms]. However, there was no main effect of attractor number alone [$\hat{\beta }$ = 14.47; *t*_(757.00)_ = 0.65; *p* = 0.51, n.s.; singular mean = 624.87 ms; plural mean = 637.37 ms]. In the adverb region (R5), there were no effects of any of the experimental manipulations (all *t*'s < 1.3).

The main clause verb region (the critical region, R6) showed a main effect of grammaticality such that ungrammatical utterances were read much more slowly than grammatical utterances [$\hat{\beta }$ = 102.56; *t*_(3657.00)_ = 6.70; *p* < 0.0001 ungrammatical mean = 651.15 ms; grammatical mean = 575.91 ms]. The main verb region also displayed an interaction of grammaticality and gender/plural type [$\hat{\beta }$ = −59.85; *t*_(1157.00)_ = −2.50; *p* = 0.01]. This appeared to be due to a larger grammaticality effect for masculine items (ungrammatical mean = 653.00 ms; grammatical mean = 567.54 ms) than for feminine items (ungrammatical mean = 653.11 ms; grammatical mean = 582.06 ms). Crucially, the main clause verb region also yielded an interaction between attractor number and grammaticality [$\hat{\beta }$ = −72.40; *t*_(3925.00)_ = −3.44; *p* = 0.0006]. Planned comparisons revealed that this was driven by an effect of attractor number in the ungrammatical conditions such that plural attractors were read more quickly than singular attractors [*t*_(103)_ = 4.48; *p* < 0.0001; plural mean = 622.48 ms; singular mean = 679.83 ms] but no difference in the grammatical conditions [*t*_(103)_ = −0.04; *p* = 0.97; plural mean = 576.09 ms; singular mean = 575.74 ms]. This agreement attraction interaction did not appear to be modulated by gender/plural type in the main clause region [$\hat{\beta }$ = 39.08; *t*_(3928.00)_ = 1.31; *p* = 0.19], though see the following section for some consideration on this finding.

Following the critical main verb, the first spillover region (R7) showed a main effect of attractor number such that plural attractor sentences were read more slowly in R7 than singular attractor sentences [$\hat{\beta }$ = 27.53; *t*_(1598.00)_ = 2.51; *p* = 0.01; plural mean = 520.22 ms; singular mean = 520.18 ms], though as the means suggest this effect is not significant in a follow-up comparison [*t*_(207)_ = 0.005; *p* > 0.99]. We believe this effect attributable to our use of dummy coding, as a sum-coded model does not reveal this effect [$\hat{\beta }$ = 0.87; *t*_(757.00)_ = 0.34; *p* = 0.74] despite qualitatively different results for all other effects. The effect of grammaticality which began at the main clause verb persisted into the first spillover region, with ungrammatical sentences read more slowly than grammatical sentences [$\hat{\beta }$ = 70.58; *t*_(3925.00)_ = 7.37; *p* < 0.0001; ungrammatical mean = 541.16 ms; grammatical mean = 499.24 ms]. Additionally, the attraction interaction of attractor number and grammaticality which began in the previous region persisted into R7 [$\hat{\beta }$ = −37.92; *t*_(3924.00)_ = −2.81; *p* = 0.005]. However, in this region this interaction was driven by significantly longer reading times to plural attractors in grammatical conditions [*t*_(103)_ = −2.37; *p* = 0.02; plural mean = 506.48 ms; singular mean = 492.01 ms]. In ungrammatical conditions, plural attractors conditioned faster reading times than singulars, though this effect did not reach significance [*t*_(103)_ = 1.58; *p* = 0.11; plural mean = 533.95 ms; singular mean = 548.36 ms]. Additionally, R7, the first spillover region, also showed a significant interaction of grammaticality and gender [$\hat{\beta }$ = −30.40; *t*_(3925.00)_ = −2.26; *p* = 0.02]. This interaction was due to significantly longer reading times to grammatical sentences with masculine attractors than those with feminine attractors [*t*_(207)_ = 4.12; *p* < 0.0001; masculine mean = 515.22; feminine mean = 486.90]. A similar trend was only marginal in the ungrammatical sentences [*t*_(207)_ = 1.77; *p* = 0.07 feminine mean = 536.89 ms; masculine mean = 553.46 ms].

Finally, in the second spillover region, there were no significant effects of any of the experimental manipulations (all *t*'s < 1.85), however the main effect of grammaticality was marginally present [$\hat{\beta }$ = 14.04; *t*_(3926.00)_ = 1.83; *p* = 0.07]. This was again because ungrammatical sentences were read longer two words downstream from the main clause verb than grammatical sentences (ungrammatical mean = 491.79 ms; grammatical mean = 477.76 ms).

#### 2.2.2.2. By gender/plural type

**Figure 2:**
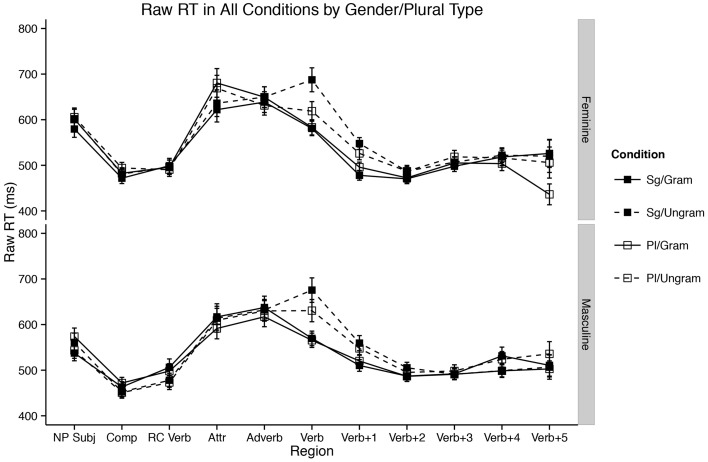
**Self-paced reading results**. Region by region means segregated by attractor number and verb number for both genders/plural types. Error bars represent the standard error of the mean computed across subject averages.

Results for the experiment segregated by plural type/gender of the attractor NP are presented in Figure 2. While our mixed-effects model presented above did not show a significant interaction of gender/plural type and the attraction effect (the three way interaction of Attractor Number × Grammaticality × Gender/Plural Type was not significant), we had two reasons for investigating the interaction further: (i) *a priori* considerations concerning the grammatical status of plural formation type in MSA (see §1.3, above) and (ii) visual inspection of the difference between the two genders in Figure 2. Specifically, we were suspicious of the possibility that feminine items were showing more attraction relative to masculine items, if the latter were indeed displaying attraction at all.

We also suspected that the lack of a significant interaction in our mixed-effects model was partially due to our choice of outlier exclusion method: Winsorizing five percent of the data could have erroneously removed long reading times to critical verbs in the Sg/Ungram and Pl/Ungram conditions—these conditions are fully ungrammatical, and since this is the first reading-time study on MSA, there was no *a priori* way to know the expected size of reading time increases to fully ungrammatical verbs. Such an interpretation is also consistent with an emerging view that agreement attraction effects are driven by reading times in the right tail of the distribution (Staub, 2009, 2010; Lago et al., 2014) It is therefore possible that a 5% cutoff by-region is too conservative and results in the exclusion of data mistaken for outliers. To this end, we ran an identical analysis with no Winsorization. The results of this analysis are qualitatively identical to the analysis presented above, save for the three-way interaction of Attractor Number × Grammaticality × Gender/Plural Type in the main clause verb region (R6); in unwinsorized model, this term emerges as marginal [$\hat{\beta }$ = 99.13; *t*_(3926.00)_ = 0.05]. This marginal effect is driven by longer reading times to Sg/Ungrammatical conditions relative to Pl/Ungrammatical conditions in the feminine items [*t*_(103)_ = 3.38; *p* = 0.001; Sg/Ungram mean = 732.05 ms; Pl/Ungram mean = 640.11 ms], a contrast which is not present for masculine items [*t*_(103)_ = 0.24; *p* = 0.81; Sg/Ungram mean = 701.74 ms; Pl/Ungram mean = 694.74].

## 3. Discussion

The results of our study clearly show that agreement attraction errors can be elicited in the comprehension of written MSA. The results from the critical verb region in this study show that reading times are universally increased in the presence of a grammatically incorrect verb, but that the magnitude of this increase in reading time is modulated by the kinds of non-subject (and therefore, structurally inaccessible for subject–verb agreement) NPs appearing in the preceding context. Specifically, when one of these preceding nouns has features which match the erroneous verb along the dimension the subject does *not* match, then a smaller increase in reading time is observed relative to cases in which no nouns in the preceding context overlap in features with the verb. Alternatively, one can view this effect as a facilitation relative to ungrammatical sentences where the attractor does *not* match the erroneously plural verb. However, it is viewed, this effect is one of the hallmarks of agreement attraction errors.

Another distinguishing feature of agreement attraction phenomena which our data reveal in MSA is the general *absence* of an analogous effect in grammatical utterances. That is, when the verb and subject agree completely in grammatical features, there is no corresponding marginal increase in reading times when a distractor NP bears distinct grammatical features—a plural NP distractor has no effect in the context of a singular subject and verb. We do observe what could be effects of this kind at the first spillover region to a small degree. However, it is worth stepping back to consider the fact that in our study, in general, effects spill over less than they do in languages such as English. We do not have an explanation for this, but note that the agreement attraction effect does not spill over in either the full items analysis or the feminine items analysis for *ungrammatical* utterances. Moreover, the magnitude is suspect: one can assess the magnitude of an attraction effect by subtracting the reading time for plural conditions from the reading time to singular conditions (see §4, below)—what Dillon et al. (2013) call the *Intrusion Effect Size*. For ungrammatical utterances, this will be a positive number (erroneous facilitation to ungrammatical verbs), whereas for grammatical utterances, this would be a negative number (erroneous inhibition to grammatical verbs). At the critical verb region, our observed intrusion effect size is 57.35 ms, whereas in the first spillover region, the observed grammatical intrusion effect is −14.47 ms. We therefore think it safe to conclude that the transient effect in the first spillover region for feminines is not a *bona fide* attraction effect in grammatical utterances. If this logic is correct, Arabic self-paced reading responses to agreement attraction configurations mirror those observed for English in Tanner et al. (2014) and Wagers et al. (2009), but not (Pearlmutter et al., 1999).

Furthermore, our results add another piece to the growing body of evidence that there is something special about the processing of plural NPs in context (Wagers et al., 2009; Tanner et al., 2014). In our data, feminine plural NPs display longer reading times than their singular counterparts in the attractor region, a finding not shared by masculine NPs (see the attractor region in Figure 2). Two explanations have been advanced for this finding in the literature: (1) that it is the result of a “plural complexity effect” insofar as it is simply more difficult to process plurals than it is to process singulars, *ceteris paribus* (Wagers et al., 2009) and (2) that it is due to a “plural integration effect” insofar as it is difficult to integrate a semantically plural NP into a context which features other singular nouns (Tanner et al., 2014, with support from findings in Nicol et al., 1997). Our findings from MSA help to shed some light on this debate. While it is possible to imagine a more nuanced version of the integration story, it is not obvious how to square the simple version of that account with the observation that semantically plural masculine/broken plural NPs do not display the reading time increase shown for feminines—both masculine and feminine plural attractors are semantically plural. If the integration explanation were correct, we might expect integration costs in both cases. While we will not attempt to resolve this fully here, we note that either one must elaborate the complexity story to include consideration of morphological plural formation strategies or return to the complexity suggestions of Wagers et al. (2009). Specifically, if one were to assume that complexity effects were correlated with the salience of plural marking on a noun (see §4, below, for some development of this idea), then we could take complexity to be about integrating plural marking with nominals stems. Alternatively, one could eschew this assumption about the salience of marking and take our data to support neither hypothesis, though we will not develop this idea here^8^.

More broadly speaking, the differences between attractor genders/plural types in both the attractor and main clause verb regions are a significant diversion from both our prediction for Arabic and the established facts for English—masculine/broken/ablauting plurals behave distinctly from feminine/sound/suffixing plurals in our data. However, one must be careful in stating *how* this difference manifests. It would be tempting to conclude that attraction occurs with feminine/sound attractors but does not occur with masculine/broken items. This conclusion, while certainly possible, must be made cautiously, as we do not have sufficient evidence in this paper to reject the idea that attraction occurs in both genders/plural types (to wit, the lack of a three-way interaction in the main clause verb region). However, at the very least one could conclude that if attraction is present in the masculine/broken/ablauting items, the effect is much smaller than it is with feminines/sound/suffixing items (**Figure 5**). Again here, the intrusion effect size is instructive: with feminines, the mean agreement intrusion effect is 68.72 ms, whereas for masculines it is 45.00, computed across subject means. While we must be agnostic as to which, one of two things is true in our data: (i) masculine/broken/ablauting items do not display attraction or (ii) they do, but to a smaller degree than feminine/sound/suffixing items.

**Figure 3:**
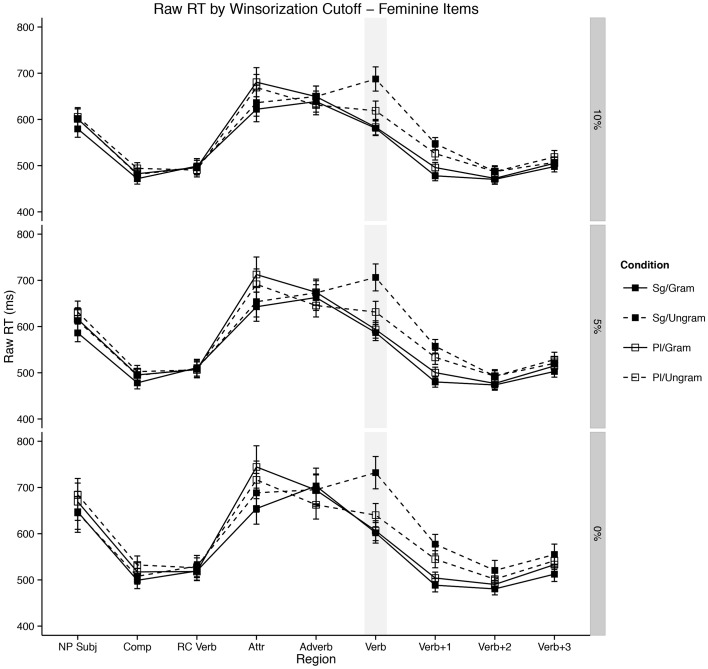
**Self-paced reading results**. Region by region means of feminine items segregated by attractor number and verb number for three Winsorization cutoff thresholds. Error bars represent the standard error of the mean computed across subject averages.

**Figure 4:**
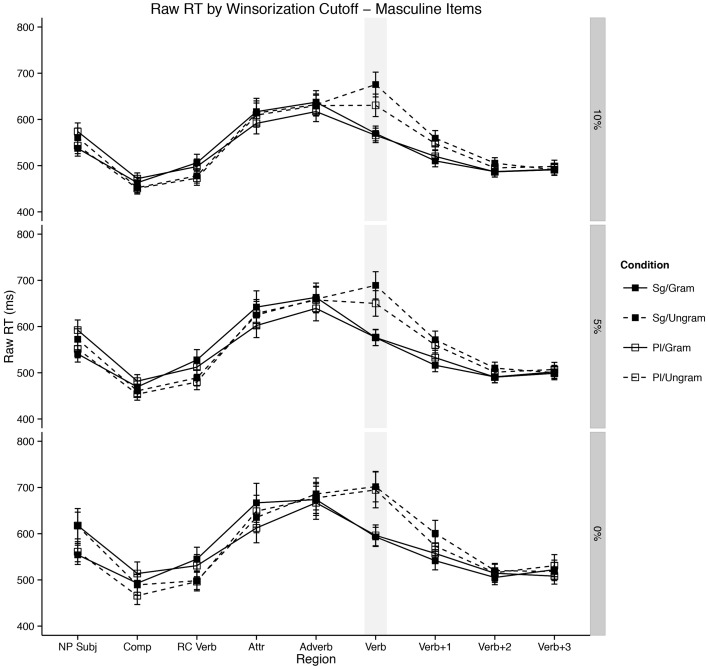
**Self-paced reading results**. Region by region means of masculine items segregated by attractor number and verb number for three Winsorization cutoff thresholds. Error bars represent the standard error of the mean computed across subject averages.

Even more broadly, we believe our results confirm a growing body of evidence in the literature about the location of agreement attraction effects in the distribution of reaction times to ungrammatical verbs (Staub, 2009, 2010; Lago et al., 2014). That is, previous work by Staub as well as Lago and colleagues has shown that the canonical pattern of agreement attraction in comprehension—facilitation to ungrammatical verbs in the presence of a matching distractor relative to ungrammatical verbs without a matching distractor—is present most strongly in the right tail of reaction time distributions to ungrammatical verbs. This appeared confirmed in our data by the change in the strength of the three-way interaction between Attractor Number, Grammaticality, and Gender as a function of our Winsorization cutoff. This can be seen for three values of Winsorization cutoffs in Figures 3, 4. In both plots, decreasing the amount of data replaced by Winsorization does not change the qualitative pattern of results anywhere except in the shaded region, the critical verb. For the feminine items (Figure 3), decreasing the amount of removed data increases the separation between the Sg/Ungram condition and the remaining three conditions. For the masculine items (Figure 4), changing the cutoff affects both the Sg/Ungram and Pl/Ungram conditions, moving the two closer together. With no cutoff, the two conditions are identical, i.e., there is no attraction present. We take this to be further evidence that the right tail of reaction time distributions is vitally important for the study of violation responses, such as those seen with agreement attraction^9^.

**Figure 5:**
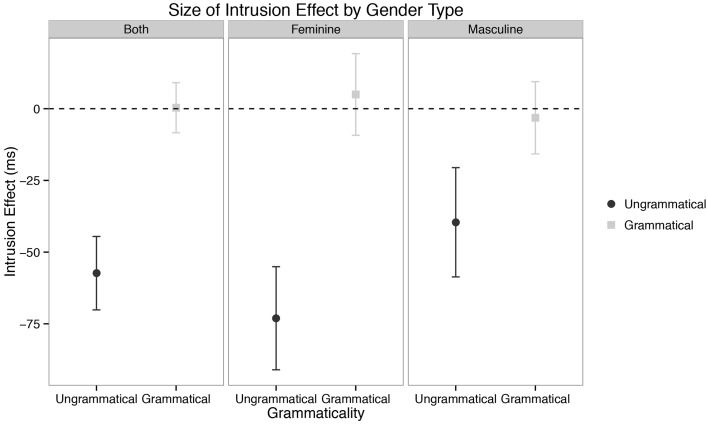
**Actual sizes of intrusion effects across different grammaticalities and gender/plural types in the self-paced reading data**. Intrusion effects are computed by subtracting retrieval times in plural attractor conditions from times in singular attractor conditions. Error bars represent the standard error of the mean computed across subject averages.

While we believe there is clearly a difference between feminine/suffixing and masculine/ablauting attractor items in our data, in this study this pluralization strategy-based difference is necessarily conflated with gender, both on the attractor NP and the verb itself. This is because of the way our stimuli were designed: all the suffixing plurals in our study were feminine nouns and all the ablauting plurals were masculine nouns. This is because of the grammar of MSA, which affords very few broken/ablauting feminine plurals which refer to animates. Moreover, grammatical case is necessarily orthographically present on masculine sound/suffixing plurals but not feminine sound plurals, making direct comparison somewhat confounded if those NPs were included.

Nevertheless, we find it plausible to tentatively assume that the differential agreement attraction effect across gender/plural type items is brought about by the different pluralization strategies and not by grammatical gender marking on the verb because of the absence of plural-based reading time trend on the attractor NPs for ablauting plurals. While it is conceivable that this lack of an effect is driven by their masculine gender, such an explanation cannot relate the *presence* of the slowdown in feminine/suffixing plurals to similar effects noted for English by Wagers et al. (2009). On the other hand, assuming the strength of the agreement attraction effect is driven by plural type allows for this cross-linguistically and theoretically coherent link as well as a unified explanation of the absence of the NP plural complexity and attraction effects.

One might reasonably wonder, at this point, what role, if any, frequency plays in explaining the observed patterns in MSA. In order to address this question, we calculated the token frequency of each attractor NP in the singular and plural form in the *Al-Hayat 1996* sub-corpus of the BYU arabiCorpus (Parkinson, 2012) and entered the plural log frequency values into our mixed effects models. In neither the attractor (R4) nor critical verb (R6) region were these terms significant in the model. However, it is worth noting that both singular [masculine mean = 1.11; feminine mean = −0.18; *t*_(39.97)_ = 6.38; *p* < 0.0001] and plural [masculine mean = 0.70; feminine mean = −1.14; *t*_(39.58)_ = 9.27; *p* < 0.0001] nouns did have significantly different log-frequencies for masculine and feminine nouns. A complete table of the frequencies for the attractor nouns in our experimental items appears in the Supplementary Materials.

The question still remains, however, as to what the explanation of this difference between the suffixing and ablauting plurals might be. Here we entertain two possibilities: (1) that the processing system does not have sufficient time for attraction effects to emerge because the system is at floor in the ungrammatical conditions and (2) there is something morphologically distinct about broken/ablauting plurals such that attraction cannot occur because the representation of number with these plurals is fundamentally different.

The first solution is plausible because broken/ablauting plurals in MSA are, on the whole, orthographically shorter than sound/suffixing plurals—usually between one and two characters shorter. Moreover, there is a clear, reliable difference between broken/ablauting and sound/suffixing plurals evident in our data set such that the latter are read around 70 ms slower than the former (see R4 Figure 2). The explanation in this approach would be that this shorter reading time is small enough that appreciable agreement attraction effects are not observable in such a short time frame—the system is simply under too much time pressure to reveal these effects and is at the *a priori* floor.

However, we do not believe this to be the correct approach for several reasons. Firstly, the directionality of this change in broken/ablauting plural reading times is in the wrong direction. Attraction in our data is revealed by the difference between plural-attractor plural-verb (Pl/Ungram) and singular-attractor plural-verb (Sg/Ungram) conditions—in both cases the plural verb is ungrammatical but only in the former case does a partially matching attractor lead to decreased reading times. However, broken/ablauting plurals clearly involve *faster* reading times across the board, meaning that the Pl/Ungram condition is undergoing an additional reading time decrease when the plural involved is broken/ablauting as opposed to when it is singular—this should increase the magnitude of the attraction effect, not decrease it. Furthermore, in our item set, the difference between mean length of plural and singular items was 1.06 characters for the feminine attractors and 0.72 characters for the masculine attractors, making it hard to specify what role a difference in mean length of 0.3 characters could be playing in a way which accounts for such a large difference between conditions.

Thus, it is reasonable to conclude that nouns with plurals formed by morphologically discontinuous CV-templates *may* drive less agreement attraction, a novel finding in sentence-level reading studies, as far as we know. The split between ablauting and suffixing lends support to the notion that *morphological marking of number* is necessary for agreement attraction to occur in Arabic. The reason for this is that—despite their decreased attraction—broken/ablauting plurals *are* still plurals semantically. Nevertheless, this semantic plurality does not contribute as much as morphological form in driving attraction rates at the critical verb region.

Two things are clear from this limited data set: (1) agreement attraction does occur with attractors in Arabic relative clauses despite the relatively inhospitable grammatical environment relative to non-clausal modifiers such as PPs (Bock and Miller, 1991) and (2) that this effect is modulated by the plural type of the attractor^10^. An immediate follow-up experiment present itself for which preparations are underway: a direct manipulate the gender of the attractor independent of number in order to confirm the argumentation that gender is not the relevant effect in this data.

## 4. Computational modeling

Since we take the procedural implementation of agreement dependency resolution to be universal, the important question thus becomes what drives language-specific differences in error profiles and what impact, if any, our findings have on theoretical explanations of agreement attraction. Here we discuss whether or not working-memory models of attraction provide a mechanism for explaining the contrast between broken/ablauting and sound/suffixing plurals seen in our data. The question is one of representation: do explicit models of agreement attraction as working memory retrieval errors provide a representational way to model the distinction between ablauting and suffixing plurals?

We answer this question by way of computational modeling in the ACT-R system of language comprehension presented by Lewis and Vasishth (2005). Given that well-specified computational models of the sentence processor exist, computational modeling can allow us to evaluate different representational commitments against the results of a system known to accurately model many aspects of working memory and sentence processing. We use ACT-R in particular because of its recent popularity in the sentence processing literature (see Lewis and Vasishth, 2005; Lewis et al., 2006; Dillon et al., 2013) and its requirement that modelers be explicit about representational commitments made for constituents in memory.

One key feature of these models that we believe is implicated by our data is the notion of activation as a zero-sum game across specified retrieval cues. In the ACT-R system, the strength of a particular retrieval cue is proportional to the logarithm of the number of items associated with that cue (Lewis and Vasishth, 2005, p. 381). Given this relationship, oneis co-construed can reduce the strength of, e.g., the number cue for a particular chunk, by removing that chunk's specification for the number cue. This in turn increases the strength of that cue for other chunks in memory which remain specified for number. The result is a reduction in the error rates and retrieval latency intrusion effect size.

In our data, one might therefore consider modeling the ablauting plural attractors with *underspecification*. Underspecification is an approach to the organization of the lexicon wherein certain grammatical features are not present at the lexical level of representation^11^. This approach would therefore remove number from the ablauting attractors and therefore increase the strength of this cue for the true subject. This is a common strategy in the ACT-R language literature for modeling disappearing and reappearing intrusion effects—see (Dillon et al., 2013) for discussion and references in the context of the difference between agreement and reflexive anaphora.

In order to test this idea with Arabic nouns, we need to make some preliminary assumptions. The first of these is that the traditional approach to Arabic grammar which organizes the lexicon in terms of consonantal roots which associate with prosodic templates (see McCarthy, 1981 for the generative approach)^12^. Furthermore, we assume that the prosodic template, despite being morphophonologically abstract, can bear grammatical information for the system—in the case of Arabic nouns, the key feature will be that the template can bear the functional load of number. Finally, we assume that the parser gives access to some form of root/template decomposition during reading, though we will remain agnostic as to the exact mechanism by which this happens.

With this background in mind, we can now ask whether underspecification of grammatical number on the template is an appropriate way to model our data from our ablauting items. Here we consider three distinct models which differ only on their representation of grammatical number as a cue to retrieval:

(9) a. A **fully-specified** model wherein number is a bivalent cue which can take two values: singular and plural
      b. A **underspecified NP** model in which nouns appearing in broken plural templates have no specification for number.
      c. A **fully-underspecified** model in which number is a fully privative cue that has only one value: plural

The fully-specified model (9a) is meant as a control, a model which accounts for the suffixing data in Arabic and against which we can compare two possible models of ablauting templates. The two models in (9b–c) are two different ways of modeling underspecification in ACT-R, and the viability of either model is the modeling result of interest.

In both the underspecified models (9b–c), underspecification is represented by the absence of a number cue on one or more constituents in memory. In the Underspecified NP model (9b), only NPs which are part of the broken/ablauting plural system lack a number cue; in the Fully Underspecified model (9c), singular verbs *also* lack a number cue. The model in (9c) therefore corresponds to a fully privative number cue system. In either of the underspecified models, representation of a sound/suffixing plural noun simply requires specifying that NP as plural.

To evaluate these models, we ran 10,000 Monte Carlo simulations in ACT-R of each of the four conditions in our experiment with each of the three models (using code first written for Badecker and Kuminiak, 2007; Badecker and Lewis, 2007). ACT-R has several free parameters which must be specified, such as the amount of activation noise present in the system. Instead of computing results across different parameter sets, these parameters were set to the most common values found in the Wong et al. (2010) Online Database of ACT-R Estimated Parameters. While this approach does not provide an argument for the robustness of our results across different parameter values, it does provide for model results using the most neutral parameter specifications.

Our interest in the ACT-R model is in the predictions it makes with respect to a retrieval event triggered at the critical verb which searches for the correct controller of agreement. The model itself provides two dependent measures of interest: (1) the rate of retrieval of each constituent chunk in memory and (2) the latency of retrieval predicted by the model. Both of these dependent measures depend on the schedule of retrievals inputted to the model, which for us included: (1) a retrieval which searches for a NP host for the relative clause at the complementizer, (2) a retrieval which searches for the subject of the embedded clause verb, and (3) a retrieval which searches for a subject of the main clause target verb. (3) is the critical retrieval for us, and all quantitative results we report concern this retrieval.

**Figure 6:**
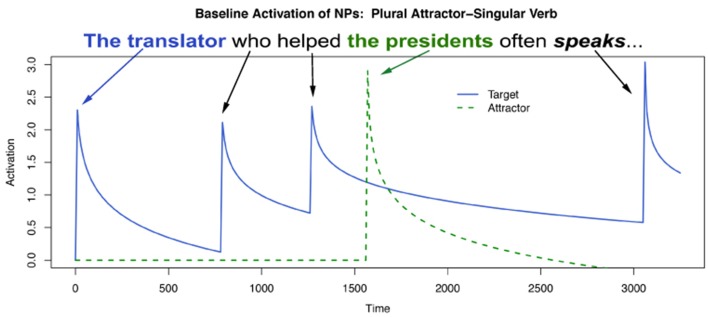
**Model activation values over time for an ACT-R model of grammatical attraction conditions**.

**Figure 7:**
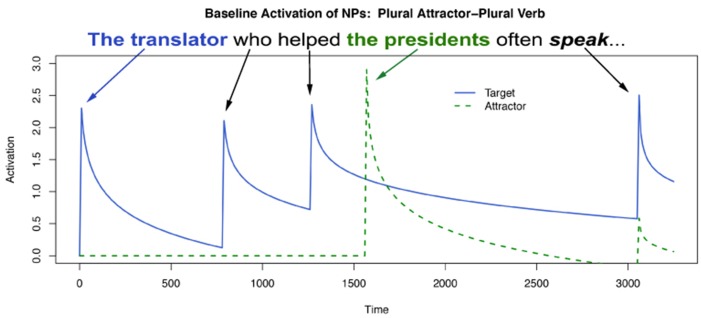
**Model activation values over time for an ACT-R model of ungrammatical attraction conditions**.

Figures 6, 7 show activation time-course plots for two of the conditions in our experiment, Pl/Gram and Pl/Ungram, in the Fully Specified model. Pl/Ungram is the attraction condition and Pl/Gram is a control insofar as it involves the same attractor/subject configuration but should yield no attraction. As can be seen in Figure 6, the retrieval triggered at the singular main clause verb involves no increased activation of the attractor, whereas when the verb is plural (Figure 7), the attractor receives a boost in activation which corresponds to the attraction effect. This is a correct result for agreement attraction insofar as the increased activation translates to a proportional increase in incorrect retrievals of the attractor.

Turning now to error rates, Table 4 shows the percentage of retrievals in which the true subject or attractor is retrieved at the main clause verb across the three model types^13^. Crucially, Table 4 shows a marked increase in error rates in the Pl/Ungram condition in the Fully Specified model; this is a predicted attraction effect. Notably, however, either of the underspecification models cause this attraction rate to fall off considerably, decreasing from 24.15 to 6.30% in the Underspecified NP model and from 24.15 to 5.88% for the fully underspecified model. Moreover, in both the underspecified models the error rate is flat across all four experimental conditions. This is a prediction of no or very little attraction effect in for broken/ablauting plurals in these models.

**Table 4:** **ACT-R Retrieval rates for the target/attractor NPs in 10,000 model runs**.

	**Condition**			
**Model Type**	**Sg/Gram**	**Sg/Ungram**	**Pl/Gram**	**Pl/Ungram**
Fully Specified	90.82/6.15%	90.90/6.29%	94.95/1.63%	74.07/24.15%
Underspecified NP	95.09/1.64%	91.07/6.53%	94.65/2.00%	90.85/6.30%
Fully Underspecified	93.90/4.63%	91.99/5.60%	93.50/4.98%	91.89/5.88%

*A fully specified model takes plurality to be a bivalent cue with possible values [SG] and [PL]; a NP underspecified model considers only ablauting plurals as underspecified for number; a fully underspecified model considers all non-plural constituents underspecified for number*.

**Figure 8:**
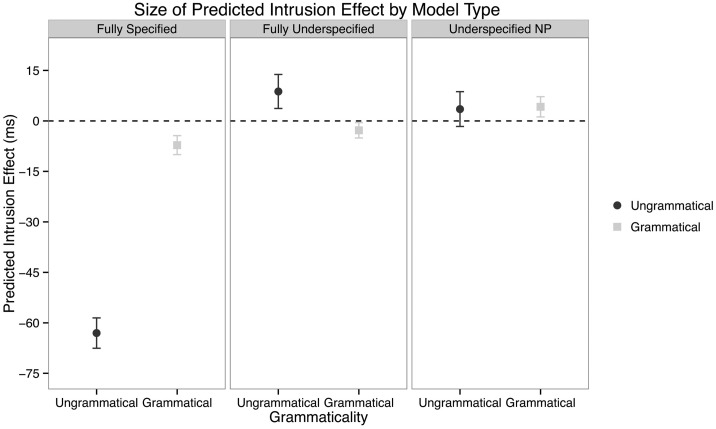
**Predicted sizes of intrusion effects for three different ACT-R models**. Intrusion effects are computed by subtracting retrieval times in plural attractor conditions from times in singular attractor conditions. Error bars represent the standard error of the mean of 10,000 Monte Carlo trials. A normal model takes number cues to be bivalent, an underspecified NP model takes number to be privative on the broken plural template NPs only, and a fully underspecified model takes number to be privative on all constituents.

Moving to predictions more analogous to our results, the ACT-R model also furnishes latencies to retrieval of any chunk, and these latencies can be used to predict the size of the agreement attraction or intrusion effect, exactly as is shown in Figure 5 for our data. Figure 8 shows the predictions of the ACT-R model across the three model types in (9). Starting with the Fully Specified Model, Figure 8 shows that the system predicts a large intrusion effect for ungrammatical utterances, exactly as we observe in our data. Turning to the two underspecification models, we observe a flattening of this effect across grammaticality. Both the Underspecified NP and Fully Underspecified models predict no obvious difference between grammatical and ungrammatical conditions.

How one views the success of these modeling results depends on one's interpretation of the empirical results in our study. If one assumes that the masculine/broken/ablauting plural attractors cause a *smaller* attraction effect than the feminine/sound/suffixing attractors, then these modeling results point to a weakness in the representational commitments or architecture of the computational model. Specifically, the dependency between number of items associated with a cue and cue strength means that the reduction in cue strength given by underspecification is all-or-nothing. What is required here is another mechanism for allowing cue strength to modulate in a continuous way—one possibility couched entirely in the memory architecture is to assume that the number cues present on broken/ablauting plurals are somehow different from the number cues present on sound/suffixing plurals in a way which leads the number cues on masculine items to be confusable (Jäger et al., 2014) with the number cue present on the main clause verb. In a system where matching is not all-or-nothing, a confusable number cue on the masculine items would lead to lower, but not completely absent, rates of attraction. We do not implement this here because of the relative novelty of the confusability proposal as well as the focus of this paper, but note that it is an intriguing possibility.

On the other hand, if one views our results as showing that masculine/broken/ablauting attractors lead to a complete *absence* of attraction effects, then our modeling exercise here can be taken to show the limited utility of underspecification in the retrieval system (but not the grammar, or the mapping between features and cues; see below) Specifically, our results would show that the model matches the data reasonably well if one assumes that underspecification is the operative difference between ablauting and suffixing plurals insofar as the former are underspecified for number. However, it is important to step back and ask why this is: it is not surprising that a model taught to ignore number (via the absence of number cues on masculines) would yield results that are invariant for plurality. The question should be whether such a state of affairs is congruent with theories of grammar or the mapping between representations used in grammar and representations used in processing^14^. This, however, is a significant shift in perspective: it requires examining the consequences of assuming that number features are not fully specified on ablauting plurals. However, this is not a innocuous assumption as it requires complicating the relationship between grammatical features and retrieval cues; we return to it in the general discussion.

## 5. General discussion

### 5.1. Universal procedural components

The results of our study suggest that agreement comprehension errors occur in Arabic similar in broad strokes to the way they occur in Slavic, Romance, and Germanic languages. Specifically, our results show that plural attractors in subject relative clauses can spuriously attract agreement in such a way that an erroneously plural verb can be read as grammatical some percentage of the time. This is an especially striking result given two properties of our stimuli which mitigate against high attraction rates: 1) the use of a distractor in a relative clause and 2) the agreeing status of the complementizer in MSA. While other studies such as Dillon et al. (2013) have demonstrated that relative clauses can still contribute to attraction at a possibly lower rate, combining the presence of these relative clauses with the disambiguating cues provided by the complementizer yields an environment where one could imagine that error rates are driven to floor or precluded altogether; nevertheless, this is not what happens in MSA.

The first property has historically been shown to drive down error rates, starting with the original study by Bock and Miller (1991) (see also Bock and Cutting, 1992). In their Experiment 2 using single-clause stimuli where the attractor was contained inside a modifying prepositional phrase, the error rate was 2.39% vs. an error rate of 1.80% for Experiment 3 using bi-clausal stimuli with the attractor inside the embedded relative clause. Note, additionally, that error rates are low across the board due to the sentence-completion task employed in that study, a task which usually results in very low error rates. There are numerous ways to model such a near-halving of the error rates, but a common approach is to assume that subject-hood or clause-mate status is relevant for cue-based retrieval—when this feature is shared by the true subject and critical verb, activation of the correct NP is boosted at the cost of the activation of the attractor NP.

This activation benefit of the true subject conferred by the clause-mate is augmented by the fact that complementizers in MSA necessarily agree in grammatical number and gender with definite NPs to which they are attached or with which the gap in the relative clause is co-construed (Ryding, 2005, pp. 322–323). If one assumes that constituent activation levels are augmented when a relative clause is attached to a head noun, then this must occur at the complementizer position in MSA. Moreover, this retrieval event necessarily includes a number (and gender) cue, unlike the equivalent retrieval event in languages without an inflecting complementizer, such as English. Thus, by the time the critical verb is encountered, more temporal decay of activation of the true subject should have occurred in English than in Arabic. This, in turn, should imply smaller error rates in Arabic than in English, since the number and gender cues on the complementizer reinforce the activation of the proper subject NP. Even in model-neutral terms, something like this is expected, since the bare fact is that the complementizer provides the speaker with additional cues to the proper subject in Arabic, but not in English.

Nevertheless, these potentially mitigating factors did not result in a complete absence of agreement attraction errors in Arabic. In both perfect and imperfect aspect, each with distinct verbal agreement affixes, attraction by the plural attractors is clearly evident in the decreased reaction time in the ungrammatical sentences with plural attractors which match the verb relative to cases where the attractor does not match the verb. Such a result is consistent with the working-memory model of agreement attraction which views the procedural underpinning of these errors as a universal part of the language comprehension system. In this model, decreased reaction times in ungrammatical sentences with matching attractors are driven by partial cue overlap between the attractor and the erroneous verb—a result which is driven by the fundamental architecture of the memory system.

### 5.2. Representing plurality

However, one marked difference between our data and those reported for other languages is the role of the morpheme which carries plural marking. Our results indicate a difference between plurals formed by suffixation and those formed by ablaut in the size or presence of the attraction effect. This is a novel observation in the agreement attraction literature for any language, as far as we are aware. In §4 we noted that the representational implications of this result for the retrieval system depend in part on the assessment of its nature: if the difference is just one of quantity, then complications could be made to the way that plurality is represented solely in the memory system (*cf*. the discussion of cue confusability). If, on the other hand, one takes the difference to be qualitative, then complications need to be in the feature-cue mapping algorithm. It is that complication which we explore in this section.

In the case where the difference between feminine/sound/suffixing and masculine/broken/ablauting plural attractors is taken to be large, then one needs to articulate the way in which grammatical features map onto retrieval cues. Recall that underspecifying a broken plural for a plural feature results in a model which matches data in which no attraction occurs in broken plural items reasonably well. However, the question would then be how to articulate the relationship between grammatical plurality and the absence of an effect of plurality in the retrieval system. One simplistic option would be to claim that semantic plurality does not not contribute to retrieval interference for plural cues expressed morphologically on the target verb. However, this simple idea is unlikely to be the entire story given the finding in the literature that semantic overlap contributes to retrieval interference elsewhere (for a close parallel to our data in English, see Bock and Eberhard, 1993; for an overview of semantic contributions to interference, see the overview and references in Van Dyke and Johns, 2012). A more nuanced view would take features from various components of formal grammar to contribute additively to cues in the retrieval system^15^.

In an additive approach, one could imagine that semantic and morphological features combined underwrite what is ultimately expressed as a plural cue in on NP chunks in memory. One could then specify that the morphology of these broken plurals contributes less to the sum that ultimately makes up plural cue values for constituents in memory such that broken plurals appear to the memory system as less plural than the sound plurals. Our results would then speak to the nature of the weights of various featural components that underwrite such an additively composite cue such that semantic plurality alone is not sufficient to yield a plural cue that causes measurable agreement attraction. Underspecification can then be seen as coherent insofar as only the morphological component of an additive cue is underspecified. This allows for a way to understand our data in a theoretically meaningful way.

More generally and independently of any one interpretation of the models of feature-cue mapping involved, the results here suggest some important conclusions about morphological representation in general and Arabic templates in particular. Regardless of the model-theoretic interpretation of these results, one fact is clear: a discontinuous and/or abstract morphological constituent modulates error rates related to plural nouns in Arabic. This is important because it underscores the morphological contribution of discontinuous alterations in form in the language. Not only is this a point which is important for language-independent theorizing, but it is a point currently under contention in the Semitic-specific priming literature. The results of this study suggest that whatever the correct representational view of the CV-template is, it must minimally be allowed to augment plurality-driven effects in reading comprehension.

## 6. Conclusion

We have demonstrated that agreement attraction errors exist in MSA in a configuration which is relatively inhospitable to the presence of such mistakes: subject relative clauses with an agreeing complementizer in a morphologically rich language. Furthermore, we showed that MSA, like English, has a plural complexity cost associated with reading suffixed plural NPs. However, we also showed that MSA differs from English in important ways concerning the nature of plural formation. Specifically, we showed that plurals formed by suffixation strongly attract agreement, whereas plurals formed by ablaut/internal vowel change do so at greatly reduced rates, if at all. Moreover, we have suggested that Arabic also provides evidence that agreement attraction effects are driven mostly by observations in the right tail of the reaction time distribution. Finally, we have provided model evidence which suggests that morphologically discontinuous plural forms in MSA require some elaboration of the way grammatical features are translated into processing cues for the memory retrieval system. Finally, we discussed how these results suggest a somewhat form-driven comprehension mechanism for agreement resolution, provided that such a model allows discontinuous form-based differences to modulate comprehension of agreement dependencies.

### Conflict of interest statement

The authors declare that the research was conducted in the absence of any commercial or financial relationships that could be construed as a potential conflict of interest.

## Footnotes

^1^In these examples and throughout the paper, the grammatical controller is in *italics*, the distractor in **bold**, and the erroneous verb in both *italic* and **bold** typeface. We do not mark ungrammatical sentences with a diacritic in this paper, as the nature of the acceptability of *prima facie* ungrammatical sentences is the object of study here.

^2^http://jimwood8.wordpress.com/2012/08/13/non-adjacent-agreement-attraction/.

^3^See any of the previously cited studies for examples of this. Note that this runs counter to the claims of some formal linguists who have occasionally treated these errors as dialectal or idiolectal variation in need of explanation (i.e., Kimball and Aissen, 1971; Kayne, 2000; Den Dikken, 2001). At the very least, one would have to maintain that the data underwriting these studies are dialectically distinct from standard English, as Kimball and Aissen (1971) do.

^4^This is actually true of a broader array of models than just the ones considered here, such as the Competition Model (e.g., MacWhinney, 1987; MacWhinney and Bates, 1989; MacWhinney, 2001).

^5^In this and all subsequent glosses, we use the following abbreviations for grammatical features: MASC = masculine gender, FEM = feminine gender, 3 = third person, PERF = perfect aspect, NOM = nominative case, ACC = accusative case, and COMP = complementizer. Finally, because Arabic orthography is ordered right-to-left, we do not gloss the Arabic itself; it is included for reference, and a gloss is included for the phonetic transcription.

^6^For both masculine and feminine nouns, the situation is modulated by definiteness, where definiteness is defined as marking with the definite article /al-/. Since all the nouns used in our study were definite, we focus on definite NPs only in this description. See (Ryding, 2005) for ample discussion.

^7^The discrepancy in gender in this sample is a product of the student body makeup at the UAEU, where the majority of testing was conducted. This university has a 3:1 female-to-male student ratio and has gender-segregated campuses. Testing was conducted on the female side because of the larger number of students, meaning that male students were not able to participate at the UAEU.

^8^An intriguing possibility, raised by a reviewer, is that the lower accuracy rates to comprehension questions in the Pl/Gram condition could be a grammatical agreement attraction effect. We cannot rule this explanation out, but note two things: first, our comprehension questions were constructed to avoid use of lexical items which underwent an experimental manipulation (such as the attractor NP and main clause verb). Therefore, such an effect would have to be driven by the main clause subject, which was invariantly singular. Second, however, not *all* of our comprehension questions asked about this subject, making it difficult to assess this hypothesis in our current data, but the idea is viable for future research.

^9^Another possibility, raised by a reviewer, is that item order matters. Concretely, the idea would be that subjects are susceptible to grammatical effects early in the experiment, with these effects diminishing over time (as the participant begins to realize what is happening in the manipulations). We agree this is a possibility in our data, but have included item order as a predictor precisely so that our experimental effects can be trusted with item order held in abeyance. We hope to return to the issue of item order more concretely in future work.

^10^One issue which we have not addressed here is the possibility raised by Gillespie and Pearlmutter (2013) that the inhospitability of relative clauses for attraction is due to lack of consideration for the semantic weight of the relative clause verb and not, say, structural or length differences between relative clauses and PPs. This interpretation is possible for our results and we hope to return to it in future work.

^11^This idea dates back to Trubetskoy's (1958) conception of the lexicon, and the modern instantiation of this notion first appears in Halle (1959).

^12^However, since our data requires no commitment to the position of vowels in this model, we will assume they are part of the prosodic template itself, *contra* (McCarthy, 1981).

^13^Note that these rates do not sum to 100 because the two NPs are not the only constituents in memory, and some small percentage of the time the system retrieves a nonsensical constituent such as a VP or CP. We ignore those results here.

^14^We thank an anonymous reviewer for forcing us to be clearer about this point.

^15^We thank an anonymous reviewer for this intriguing suggestion.
