# Supplementary material for: Representing number in the real-time processing of agreement: self-paced reading evidence from Arabic
Source: Front Psychol. 2015 Apr 9;6:347. doi: 10.3389/fpsyg.2015.00347 (PMC4390991; doi:10.3389/fpsyg.2015.00347)
Supplement: Supplementary file 1 [file Table1.PDF]

Frontiers Psychology

Original Research
March 25, 2015

Supplementary Material: Representing number in the real-time processing of agreement: Self-paced reading evidence from Arabic

Matthew A. Tucker [1,*], **Ali Idrissi** [2] **and Diogo Almeida** [1]

[1] *Language, Mind, and Brain Laboratory, Department of Psychology, Science Division, New York University Abu Dhabi, Abu Dhabi, United Arab Emirates*

[2] *Department of English Literature and Linguistics, Qatar University, Doha, Qatar*

Correspondence*:
Matthew A. Tucker
NYUAD (A2-166B), P.O. Box 129188, Abu Dhabi, United Arab Emirates,
matt.tucker@nyu.edu

Encoding and navigating linguistic representations in memory

1 SUPPLEMENTARY MATERIALS

1.1 EXPERIMENTAL MATERIALS

(1) a. المترجم الذي ساعد الرئيس أحياناً يتكلم خمس لغات بفصاحة.
b. The translator who worked for the president(s) occasionally speak(s) five languages fluently.

(2) a. الطالب الذي رأى الأستاذ بالأمسِ درس الهندسة الكهربائية في الجامعة.
b. The student who saw the professor(s) yesterday studied electrical engineering at the university.

(3) a. الأستاذ الذي علّم الطالب جيداً عمل كمذيع في التلفزيون.
b. The teacher who taught the student well worked as a host in television.

(4) a. التاجر الذي درّب العامل كثيراً تعلم اللغة الانجليزية في بريطانيا .
b. The businessman who trained the workers a lot learned English in the UK.

(5) a. المهندس الذي استقبل العالِم بالصدفةِ يعمل على ابتكار جديد.
b. The engineer who met the scientist by chance is working on a new invention.

(6) a. الشرطي الذي اعتقل اللص بسرعةٍ يساعد المارة على عبور الشارع.
b. The policeman who convicted the thief quickly helps the pedestrians in crossing the street.

(7) a. المحامي الذي أربك الشاهد بدهاءٍ ردع التهم عن موكله في المحكمة.
b. The lawyer who startled the witness cunningly stopped the accusation of his client in the court.

(8) a. الطباخ الذي وبّخ النادل بشدةٍ يشتغل في مطعم غالٍ خلال الصيف.
b. The cook who scolded the waiter forcefully works in an expensive restaurant during the summer.

(9) a. اللاعب الذي عارض الحَكم بغضبٍ فاز بجائزة أفضل لاعب.

b. The (football) player who admonished the referee angrily won the prize of the best player.

(10) a. العامل الذي ساعد الجندي بالأمسِ يَسوق شاحنة كبيرة لفائدة الشركة.

b. The worker who helped the soldier yesterday drives a large truck for the company.

(11) a. الفنان الذي خدم الملك بتفانٍ تبرع بلوحة لسفير الولايات المتحدة.

b. The artist who served the king devotedly gave a portrait to the ambassador of the United States.

(12) a. المذيع الذي قدّم الشيخ ببراعةٍ قابل الوزير في حفل رأس السنة.

b. The host who presented the sheikh skillfully met with the minister at the New Year's party.

(13) a. المحلل الذي نصح الوزير بذكاءٍ يتناول القضية الفلسطينية بعمق.

b. The analyst who advised the minister intelligently discusses the Palestinian issue in depth.

(14) a. الطفل الذي رأى الأمير سلفاً يزور العائلة الملكية كل أسبوع.

b. The child who saw the prince before visits the royal family each week.

(15) a. الشرطي الذي استجوب القاتل بجديّةٍ سأل المشاة عن سبب الجريمة.

b. The policeman who questioned the murderer seriously asked the pedestrians about the reasons for the crime.

(16) a. المعلم الذي علّم الطفل بتفانٍ حضر حفل تخرج الطلاب.

b. The teacher who taught the child dedicatedly attended the graduation party of the students.

(17) a. المجرم الذي هاجم الولد بشراسةٍ يخترق نقطة التفتيش كل ليلة.

b. The criminal who attacked the boy viciously breaks through the checkpoint every night.

(18) a. المهاجر الذي حدّث الزائر طويلاً يشعر بالحنين لوطنه دائما .

b. The emigrant who spoke with the visitor for a long time feels nostalgic for his country always.

(19) a. المستشار الذي حذّر الرئيس بالأمسِ وجد الحل للمشكلة المالية.

b. The consultant who warned the president yesterday found a solution for the financial problem.

(20) a. السائق الذي رافق السفير بانتظامٍ يعمل سبعة أيام في الأسبوع.

b. The driver who accompanied the ambassador regularly works seven days a week.

(21) a. الطيار الذي حيّا الضيف بحرارةٍ سأل أسئلة كثيرة خلال الرحلة.

b. The pilot who greeted the guest warmly asked many questions during the flight.

(22) a. السجّان الذي عذّب المسجون باستمرارٍ ينظف الزنازنة كل ليلة.

b. The jailer who tortured the prisoner constantly cleans the cells nightly.

(23) a. النجار الذي شغّل الصانع باحترامٍ أعدّ قطع أثاث رائعة للمعرض.

b. The carpenter who employed the craftsman respectfully made wonderful furniture for the exhibition.

(24) a. الطبيب الذي عالج الضابط بسرعةٍ ينخرط في الجيش الوطني.

b. The doctor who healed the officer quickly is joining into the national army.

(25) a. الممرضة التي عالجت المريضة بعنايةٍ تدرس في مستشفى الجامعة.

b. The nurse who is treating the patient carefully studies at the university hospital.

(26) a. الملكة التي ولدت الأميرة حديثاً تظهر في العلن كل أسبوع.

b. The queen who gave birth to the princess recently appears in public every week.

(27) a. البائعة التي شكرت الزبونة بحماسٍ فرحت بالفائدة الكبيرة.

b. The seller who thanked the customer enthusiastically was happy with the large profit.

(28) a. الكاتبة التي وصفت الحسناء بدقةٍ تبيع كتبا كثيرة للجمهور.

b. The novelist who described the beautiful (woman) accurately sells many books to the public.

(29) a. المدربة التي اهتمت باللاعبة جداً اشتغلت في الأكاديمية الوطنية للمبارزة.

b. The coach who was very interested in the player worked at the National Fencing Academy.

(30) a. القابلة التي اعتنت بالفتاة تكراراً تتطوع في مستشفى الجامعة.

b. The midwife who cared for the girl repeatedly volunteers at the university hospital.

(31) a. الجندية التي قابلت الشرطية بالأمسِ أحبّت الشغل في القاعدة الجوية.

b. The soldier (fem.) who met the policewoman yesterday loved the atmosphere at the air base.

(32) a. المطربة التي استقبلت الراقصة سابقاً تغني مع أوركسترا المدينة.

b. The singer who met the dancer previously sings with the city orchestra.

(33) a. المربية التي ربّت الطالبة بحنانٍ سافرت إلي بلدها الأم.

b. The nanny who cared for the schoolgirl affectionately returned to her home country.

(34) a. المخرجة التي اختارت الممثلة بشغفٍ تنتج فيلما كل شهر.

b. The producer who enthusiastically chose the actress produces a film every month.

(35) a. المصورة التي صورت الساحرة بتفننٍ نشرت الصور في كتاب جديد.

b. The photographer who photographed the witch artistically published the photos in a new book.

(36) a. الخادمة التي ساعدت المربية بجدٍّ تنظف الغرف سبعة أيام في الأسبوع.

b. The maid who helped the nanny earnestly cleans the rooms seven days a week.

(37) a. المذيعة التي دعت المؤرّخة بلطفٍ قدمت برنامجاً في التلفيزيون.

b. The announcer who invited the historian nicely presented a program on TV.

(38) a. العارضة التي التقت بالمحاسِبة تكراراً تملك كثيرا من الملابس الغالية.

b. The model who met the accountant repeatedly owns a lot of expensive clothes.

(39) a. المساعِدة التي خدمت الصيدليّة بإخلاصٍ جمعت كل التقارير القديمة.

b. The assistant who served the pharmacist loyally collected all of the old reports.

(40) a. الصحفية التي قابلت الفائزة صدفةٍ تكتب في صحف كثيرة.

b. The journalist who interviewed the winner by chance writes in many newspapers.

(41) a. البدوية التي زارت المزارعة ليلاً تسكن في وسط الصحراء.

b. The bedouin who visited the farmer at night lives in the middle of the desert.

(42) a. الطبيبة التي عالجت الطفلة مؤخراً اكتشفت شفاء للمرض الرهيب.

b. The doctor who treated the girl recently discovered a cure for the terrible disease.

(43) a. الفنانة التي راسلت الناشرة بشغفٍ رغبت في عقد جديد.

b. The artist who corresponded with the publisher eagerly desired a new contract.

(44) a. التلميذة التي أُعجبت بالشاعرة بشدةٍ قرأت قصائد كثيرة العام الماضي.

b. The student who admired the poet strongly read many poems last year.

(45) a. المديرة التي اتصلت بالمؤلفة نهاراً تشرف على كثيرٍ من المشاريع الكبيرة.

b. The editor who contacted the author during the day supervises many large projects.

(46) a. العميدة التي استدعت الأستاذة بغضبٍ لاحظت خللا في أقسام الجامعة.

b. The dean who summoned the professor angrily observed a problem in the university departments.

(47) a. الموسيقيّة التي رافقت المغنية بمهنيّةٍ عزفت مع الفرقة الوطنية للموسيقى.

b. The musician who accompanied the singer professionally played with the national music group.

(48) a. السفيرة التي استضافت المندوبة سنوياً تحدثت في الأمم المتحدة.

b. The ambassador who hosted the diplomat yearly spoke at the United Nations.

2 SUPPLEMENTARY ANALYSIS MATERIALS

2.1 LMEMS

This section contains supplementary tables for linear mixed effects models not reported in the main text. Tables 1–3 present the model details for the adverb and two post-critical spillover regions. As stated in the main text, these models were fit using restricted maximum likelihood estimation using seven core predictors:

(49) Predictors for Mixed Effects Models:

a. **Attr**(actor Number): the grammatical number of the relative clause object/distractor (default value: singular)
b. **Grammaticality**: whether or not the target verb matches the true subject (default value: grammatical)
c. **Gender/Plural Type**: the gender of the relative clause object/attractor (default value: feminine/sound/suffixing)
d. **Item Order**: the position in the randomization of items which the experimental item appeared
e. **Frequency**: the frequency of the relative clause object/attractor noun
f. **Length**: the length, in characters, of the word in the region being analyzed
g. **Previous Region RT**: the immediately previous region's reading time

The predictors in (49a–c) were categorical, whereas the others were continuous. In addition to the predictors in (49), we also included interaction terms for each of the categorical predictors. One particular feature of these models worth keeping in mind is the fact that *frequency* does not refer to each word's frequency, but rather that of the attractor NP. This was done because corpus solutions for Arabic are somewhat limited, and it was not feasible to include *every* word's frequency in the models. All models treated both subjects and items as random effects by including random intercepts for both. For hypothesis testing, we computed degrees of freedom for a t-test via the Welch-Satterthwaite approximation in R (**R Core Team**, 2014) using the `lmerTest` package (**Kuznetsova et al.**, 2014).

We assessed the validity of applying the suggestions of **Barr et al.** (2013) to fit models with maximal random effects structures including random slopes. However, any model other with an articulated random effects structure over and above random intercepts for subjects and items failed to converge in our mixed modeling of the comprehension question results. Since we were interested in keeping the random effects structures parallel across both comprehension question and reading times models, we fit models with random intercepts for subjects and items only in all models reported here and in the main text.

Supplementary Table 1. Table of coefficients for a linear mixed effects regression with gender/plural type for the adverb region (R5). p-values computed using the Welch-Satterthwaite approximation. Predictors significantly different from 0 at $\alpha = 0.05$ highlighted in bold.

Factor	β	t	df	p
**Intercept**	**614.57**	**13.69**	**86.00**	**<0.0001**
Attr	-7.60	-0.42	1663.00	0.67
Grammaticality	15.10	0.94	3925.00	0.35
Gender/Plural Type	12.38	0.54	156.00	0.59
**Item Order**	**-1.36**	**-18.31**	**3969.00**	**< 0.0001**
**Attr Frequency**	**-19.56**	**-2.25**	**113.00**	**0.03**
**Length**	**16.73**	**2.57**	**51.00**	**0.01**
**Previous Region RT**	**0.08**	**8.81**	**4066.00**	**< 0.0001**
Attr × Gram	-29.19	-1.29	3926.00	0.20
Attr × Gender	-6.72	-0.30	3930.00	0.77
Gram × Gender	-16.61	-0.74	3932.00	0.46
Attr × Gram × Gender	42.24	1.32	3928.00	0.19

Supplementary Table 2. Table of coefficients for a linear mixed effects regression with gender/plural type for the first spillover region (R7). p-values computed using the Welch-Satterthwaite approximation. Predictors significantly different from 0 at $\alpha = 0.05$ highlighted in bold.

Factor	β	t	df	p
**Intercept**	**487.00**	**27.21**	**169.00**	**< 0.0001**
**Attr**	**27.53**	**2.51**	**1598.00**	**0.01**
**Grammaticality**	**70.58**	**7.37**	**3925.00**	**< 0.0001**
Gender/Plural Type	9.21	0.60	124.00	0.55
**Item Order**	**-0.98**	**-21.91**	**3955.00**	**< 0.0001**
Attr Frequency	5.31	0.92	145.00	0.36
**Length**	**17.63**	**6.35**	**43.00**	**< 0.0001**
**Previous Region RT**	**0.02**	**4.04**	**4053.00**	**< 0.0001**
**Attr × Gram**	**-37.92**	**-2.81**	**3924.00**	**0.005**
Attr × Gender	-11.69	-0.86	3924.00	0.39
**Gram × Gender**	**-30.40**	**-2.26**	**3925.00**	**0.02**
Attr × Gram × Gender	23.24	1.22	3927.00	0.22

3 SUPPLEMENTARY FREQUENCY COUNTS

Frequency effects were evaluating by calculating the raw frequency per 100,000 words in the *Al-Hayat 1996* sub-corpus of the BYU arabiCorpus (**Parkinson**, 2012). The arabiCorpus was chosen because it is the only freely available corpus of Arabic which provides part of speech-restricted searching and normalized frequency counts. However, the arabiCorpus is quite large *in toto* — 173,600,000 words — and contains a somewhat slow search function. In order to reduce search times, the authors of that corpus suggest restriction to a sub-corpus unless frequency counts in sub-corpora return zero. *Al Hayat 1996* is the largest such sub-corpus, comprised of 21,564,239 words from the 1996 edition of *Al-Hayat*, a leading pan-Arab newspaper published in London.[1] Searches were conducted for both the singular and plural form of all attractor NPs in our study, though only the plural frequencies are used in the mixed effects models we

[1] `http://alhayat.com/`.

Supplementary Table 3. Table of coefficients for a linear mixed effects regression with gender/plural type for the second spillover region (R8). p-values computed using the Welch-Satterthwaite approximation. Predictors significantly different from 0 at $\alpha = 0.05$ highlighted in bold.

Factor	β	t	df	p
**Intercept**	**483.12**	**31.80**	**190.00**	**< 0.0001**
Attr	-0.08	-0.01	1678.00	0.99
Grammaticality	14.04	1.83	3926.00	0.07
Gender/Plural Type	13.53	1.12	146.00	0.27
**Item Order**	**-0.85**	**-23.65**	**3951.00**	**< 0.0001**
Attr Frequency	-4.56	-0.94	177.00	0.35
**Length**	**8.64**	**5.03**	**47.00**	**< 0.0001**
**Previous Region RT**	**0.05**	**8.45**	**4028.00**	**< 0.0001**
Attr × Gram	1.88	0.17	3926.00	0.86
Attr × Gender	-0.89	-0.08	3926.00	0.94
Gram × Gender	1.24	0.12	3926.00	0.91
Attr × Gram × Gender	-8.59	-0.56	3928.00	0.57

report in the paper. All searches were conducted in accordance with the suggestions of the arabiCorpus authors. The complete list of frequencies appears in Table 4.

4 SUPPLEMENTARY CODE/DATA

The raw data (including filler items) and R code used to process these data are available at `http://figshare.com/articles/Data_for_Representing_number_in_real_time_processing_Self_paced_reading_evidence_from_Arabic_/1332420` and/or can be obtained by contacting the corresponding author.

Supplementary Table 4. Raw and frequency counts per 100,000 words for all attractor NPs in experimental materials.

Item	Singular	Singular Frequency	Plural	Plural Frequency
1	الرئيس	315.14	الرؤساء	10.85
2	الأستاذ	10.3	الأساتذة	4.54
3	الطالب	27.29	الطلاب	9.21
4	العامل	23.84	العمال	16.54
5	العالِم	126.42	العلماء	10.93
6	اللصَ	1.09	اللصوص	0.82
7	الشاهد	6.64	الشهود	4.54
8	النادل	0.2	الندل	0.13
9	الحَكم	71.76	الحكام	6.19
10	الجندي	1.63	الجنود	11.27
11	الملك	33.63	الملوك	3.2
12	الشيخ	52.3	الشيوخ	6.28
13	الوزير	137.46	الوزراء	86.37
14	الأمير	36.14	الأمراء	1.89
15	القاتل	3.24	القُتال	7.47
16	الطفل	8.81	الأطفال	16.58
17	الولد	12.31	الأولاد	5.11
18	الزائر	3.92	الزوار	5.03
19	الرئيس	315.14	الرؤساء	10.85
20	السفير	30.89	السفراء	3.94
21	الضيف	4.18	الضيوف	2.11
22	المسجون	0.61	السجناء	2.09
23	الصانع	6.15	الصُناع	1.77
24	الضابط	5.83	الضباط	6.73
25	المريضة	0.44	المريضات	0.01
26	الأميرة	1.97	الأميرات	0.52
27	الزبونة	0.04	الزبونات	0.02
28	الحسناء	0.65	الحسناوات	0.09
29	اللاعبة	0.6	اللاعبات	0.46
30	الفتاة	4.84	الفتيات	1.35
31	الشرطية	0.19	الشرطيات	0.01
32	الراقصة	0.85	الراقصات	0.34
33	الطالبة	1.26	الطالبات	0.49
34	الممثلة	4.39	الممثلات	0.41
35	الساحرة	0.57	الساحرات	0.06
36	المربية	0.18	المربيات	0.08
37	المؤرّخة	0.33	المؤرّخات	0.01
38	المحاسبة	2.8	المحاسبات	0.1
39	الصيدلَيّة	0.3	الصيدلَيّات	0.19
40	الفائزة	1.25	الفائزات	0.06
41	المزارعة	0.1	المزارعات	0.001
42	الطفلة	1.89	الطفلات	0.03
43	الناشرة	0.07	الناشرات	0.01
44	الشاعرة	1.33	الشاعرات	0.1
45	المؤلفة	2.96	المؤلفات	3.0
46	الأستاذة	0.83	الأستاذات	0.01
47	المغنية	0.86	المغنيات	0.06
48	المندوبة	0.59	المندوبات	0.03

REFERENCES

Barr, D. J., Levy, R., Scheepers, C., and Tily, H. J. (2013), Random effects structure for confirmatory hypothesis testing: Keep it maximal, *Journal of Memory and Language*, 68, 3, 255–278

Kuznetsova, A., Bruun Brockhoff, P., and Haubo Bojesen Christensen, R. (2014), lmerTest: Tests for Random and Fixed Effects for Linear Mixed Effects Models

Parkinson, D. B. (2012), ArabiCorpus, Available online at `http://arabicorpus.byu.edu/`

R Core Team (2014), R: A Language and Environment for Statistical Computing, R Foundation for Statistical Computing, Vienna, Austria
